# Supplementary material for: Orbital hybridization of donor and acceptor to enhance the conductivity of mixed-stack complexes
Source: Nat Commun. 2024 Apr 16;15:3028. doi: 10.1038/s41467-024-47298-1 (PMC11021477; doi:10.1038/s41467-024-47298-1)
Supplement: Supplementary file 1 — Supplementary Information [file 41467_2024_47298_MOESM1_ESM.pdf]

Supplementary Information

Orbital hybridization of donor and acceptor to enhance the conductivity of mixed-stack complexes

Tomoko Fujino,*[1] Ryohei Kameyama,[1] Kota Onozuka,[1] Kazuki Matsuo,[1] Shun Dekura,[1] Tatsuya Miyamoto,[2] Zijing Guo,[2] Hiroshi Okamoto,[2] Toshikazu Nakamura,[3] Kazuyoshi Yoshimi,[1] Shunsuke Kitou,[2] Taka-hisa Arima,[2,4] Hiroyasu Sato,[5] Kaoru Yamamoto,[6] Akira Takahashi,[7] Hiroshi Sawa,[8] Yuiga Nakamura,[9] Hatsumi Mori*[1]

[1]The Institute for Solid State Physics, The University of Tokyo, 5-1-5 Kashiwanoha, Kashiwa, Chiba 277-8581, Japan

[2]Department of Advanced Materials Science, The University of Tokyo, 5-1-5 Kashiwanoha, Kashiwa, Chiba 277-8561, Japan

[3]Institute for Molecular Science, 38 Nishigo-Naka, Myodaiji, Okazaki, Aichi 444-8585, Japan

[4]RIKEN Center for Emergent Matter Science (CEMS), Wako 351-0198, Japan

[5]Rigaku Corporation, 3-9-12 Matsubara, Akishima, Tokyo 196-8666, Japan

[6]Department of Physics, Okayama University of Science, 1-1 Ridaicho, Kita-ku, Okayama 700-0005, Japan

[7]Graduate School of Engineering, Nagoya Institute of Technology, Gokiso-cho, Showa-ku, Nagoya, Aichi, 466-8555 Japan

[8]Department of Applied Physics, Nagoya University, Furo-cho, Chikusa-ku, Nagoya 464-8603, Japan

[9]Japan Synchrotron Radiation Research Institute (JASRI), SPring-8, 1-1-1, Kouto, Sayo-cho, Sayo-gun, Hyogo 679-5198, Japan

***Corresponding author**

Email: fujino@issp.u-tokyo.ac.jp (Tomoko Fujino) and hmori@issp.u-tokyo.ac.jp (Hatsumi Mori)

Table of Contents

1. Structures of relevant donors and acceptors **4**

Supplementary Fig. 1: Structures of donors and acceptors involved in mixed-stack complexes........ 4

2. Synthesis **4**

Supplementary Note 1: General synthetic procedure. 4

Supplementary Note 2: Material sources. 5

Supplementary Note 3: Synthesis of donor **2S** and charge-transfer salt **2S**•BF_4. 5

Supplementary Fig. 2: Synthesis of **2S**•BF_4. 6

3. Electrochemical properties **6**

Supplementary Note 4: Cyclic voltammetry measurements. 6

Supplementary Fig. 3: Cyclic voltammograms of donors and acceptors. 7

4. Theoretical calculations for isolated molecules **7**

Supplementary Note 5: Calculation of molecular structures. 7

Supplementary Fig. 4: Geometry and molecular orbitals of neutral **2S**. 8

Supplementary Fig. 5: Calculated SOMO shapes of donors in a radical cation form and acceptors in a radical anion form. 9

Supplementary Fig. 6: Calculated energy levels of orbitals for neutral donors and acceptors. 9

5. Single-crystal structures **10**

Supplementary Note 6: Single-crystal XRD measurements 10

Supplementary Table 1: Crystallographic data for single-crystal **2S** and **2S**•BF_4. 12

Supplementary Fig. 7: Single-crystal structure of **2S** at 293 K.. 13

Supplementary Fig. 8: Single-crystal structure of **2S**•BF_4 at 293 K. 13

Supplementary Table 2: Crystallographic data for single-crystal mixed-stack complexes. 14

Supplementary Fig. 9: Single-crystal structures of mixed-stack complexes at 300 K. 15

Supplementary Fig. 10: Symmetry elements in the single-crystal structure of mixed-stack complexes at 300 K. 15

Supplementary Fig. 11: Single-crystal structures of donors in mixed-stack complexes at 300 K. 16

Supplementary Fig. 12: Single-crystal X-ray diffraction precession image of the 0 *K L* plane in the reciprocal lattice of **2S–F_4** at 300 K. 16

Supplementary Fig. 13: Single-crystal X-ray diffraction precession image of the 0 *K L* plane of **2S–F_4** at 200 K. 17

Supplementary Fig. 14: Single-crystal X-ray diffraction precession images of the *H* 0 *L* (a) and 3 *K L* (b) planes in the reciprocal lattice of **2S–F_4** at 300 K. 17

Supplementary Fig. 15: Single-crystal X-ray diffraction precession image of the *H* 0 *L* plane in the

reciprocal lattice of **2S–F$_4$** at 200 K.......... 18

Supplementary Fig. 16: Temperature-dependence of single-crystal X-ray diffraction precession of **2S–F$_4$**.......... 18

Supplementary Fig. 17: Possible Models proposed for the molecular arrangement of **2S–F$_4$** superlattice observed at 200 K... 19

6. Theoretical calculations **19**

Supplementary Note 7: Band and crystal orbital calculations.......... 19

Supplementary Fig. 18: Band structures of mixed-stack complexes.......... 21

Supplementary Fig. 19: Crystal orbitals of mixed-stack complexes. 22

Supplementary Fig. 20: Wannier interpolation bands and band dispersion.......... 23

Supplementary Fig. 21: The maximally localized Wannier function of **2O–F$_4$** in a cell. 24

Supplementary Fig. 22: The maximally localized Wannier function of **2O–F$_2$** in a cell. 24

Supplementary Fig. 23: The maximally localized Wannier function of **2O–F$_2$** in a cell. 24

Supplementary Fig. 24: The maximally localized Wannier function of **2S–F$_4$** in a cell. 25

Supplementary Fig. 25: The maximally localized Wannier function of **2S–F$_2$** (major occupancy) in a cell.......... 25

Supplementary Fig. 26: The maximally localized Wannier function of **2S–F$_2$** (minor occupancy) in a cell.......... 25

Supplementary Fig. 27: Labels for *t* values for mixed-stack complexes.......... 26

Supplementary Table 3: Transfer integrals for mixed-stack complexes.. 27

Supplementary Fig. 28: Effective direct Coulomb interactions in a cell of **2O–F$_4$**.......... 27

Supplementary Fig. 29: Effective direct Coulomb interactions in a cell of **2O–F$_2$** in the major occupancy.......... 28

Supplementary Fig. 30: Effective direct Coulomb interactions in a cell of **2O–F$_2$** in the minor occupancy.......... 28

Supplementary Fig. 31: Effective direct Coulomb interactions in a cell of **2S–F$_4$**.......... 29

Supplementary Fig. 32: Effective direct Coulomb interactions in a cell of **2S–F$_2$** in the major occupancy.......... 29

Supplementary Fig. 33: Effective direct Coulomb interactions in a cell of **2S–F$_2$** in the minor occupancy.......... 30

7. Optical properties.......... **30**

Supplementary Note 8: Optical reflection spectroscopy measurements 30

Supplementary Fig. 34: Reflectivity spectra of mixed-stack complexes. 32

Supplementary Fig. 35: Temperature-dependent polarized reflectivity spectra. 33

Supplementary Table 4: The fundamental molecular vibration modes of donor and acceptor molecules estimated in the relevant ionicity calculated from their neutral and ionic states. 34
Supplementary Table 5: Parameters used in the calculation of the vibronic spectrum of **2S–F$_4$**.......34
8. Electrical conductivities..35
Supplementary Note 9: Electrical resistivity measurements 35
Supplementary Fig. 36: *I–V* curve of mixed-stack complexes. 35
9. Magnetic properties...36
Supplementary Note 10: ESR measurements..36
Supplementary Fig. 37: Temperature-dependent ESR spectra of **2S–F$_4$**...37
Supplementary Fig. 38: Enlarged ESR spectra of **2S–F$_4$**. 38
Supplementary Fig. 39: Parameters for ESR spectra of mixed-stack complexes...............................39
Supplementary Note 11: Static magnetic susceptibility measurements 40
Supplementary Fig. 40: Temperature-dependent susceptibility of mixed-stack complexes................40
10. NMR spectra...41
Supplementary Fig. 41: ^{1}H NMR spectrum of **2S** in $CDCl_3$. 41
Supplementary Fig. 42: ^{13}C NMR spectrum of **2S** in $CDCl_3$. 42
11. Coordinates of optimized structures..43
Supplementary Table 6: The geometry of optimized structure for neutral **2S**. 43
Supplementary Table 7: The geometry of neutral **2S** with constrained torsional angle $|\theta_1|$ to be 180°. 44
Supplementary Table 8: The geometry of optimized structure for radical cation **2S$^{•+}$**. 46
Supplementary Table 9: The geometry of optimized structure for neutral **F$_4$**...................................47
Supplementary Table 10: The geometry of optimized structure for radical anion **F$_4^{•−}$**.......................48
Supplementary Table 11: The geometry of optimized structure for neutral **F$_2$**..................................49
Supplementary Table 12: The geometry of optimized structure for radical anion **F$_2^{•−}$**.......................50
12. Supplementary references...51

1. Structures of relevant donors and acceptors

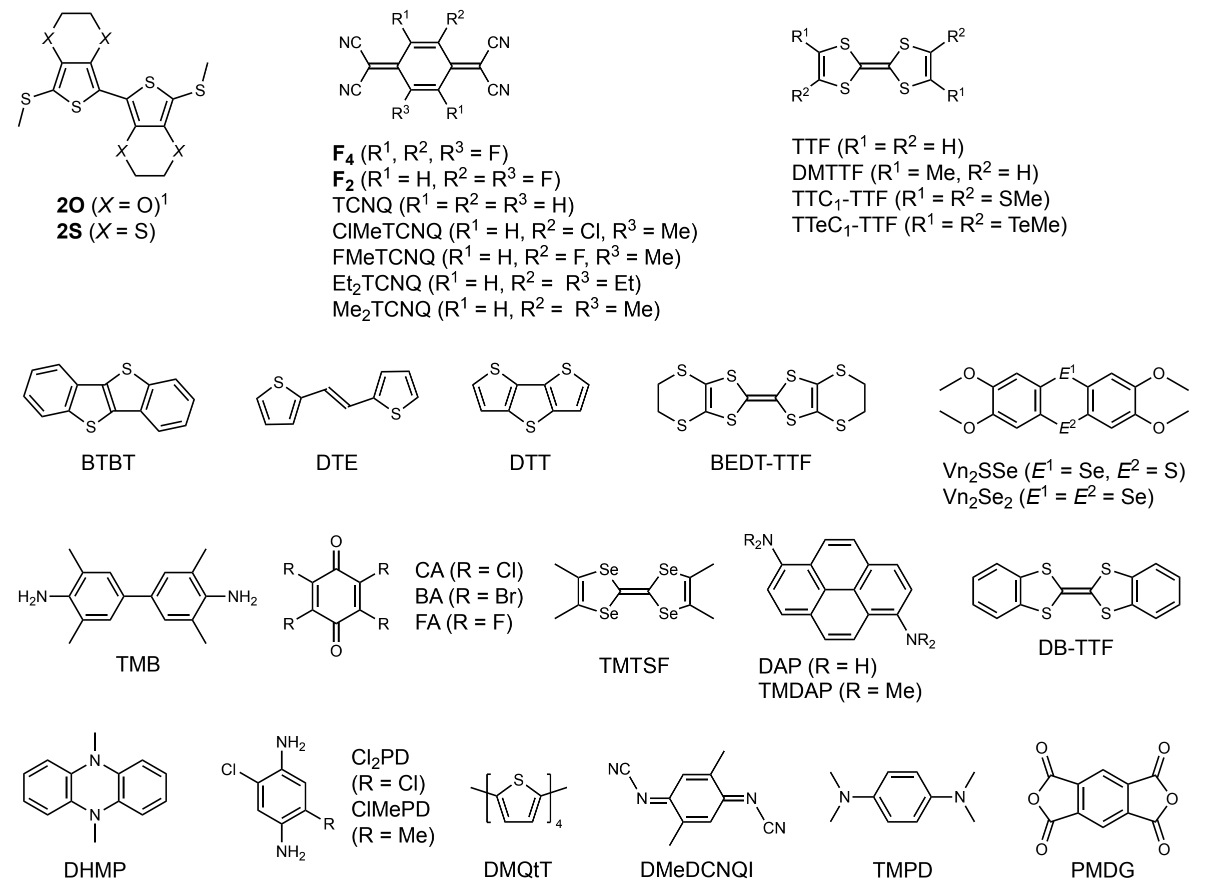

Supplementary Fig. 1: Structures of donors and acceptors involved in mixed-stack complexes. The Combinations of neutral donors[1] and acceptors are shown in Figs. 1b and 2.

2. Synthesis

Supplementary Note 1: General synthetic procedure.

All the reactions were carried out under an inert atmosphere of argon except for chemical oxidation reactions for the synthesis of mixed-stack complexes. Analytical thin-layer chromatography (TLC) was performed on a glass plate coated with silica gel (230–400 mesh, 0.25 mm thickness) containing a fluorescent indicator (silica gel $60F_{254}$, Merck). Preparative GPC was performed on JAIGEL 1HR and 2HR polystyrene columns (Japan Analytical Industry Co., Ltd.) with chloroform as the eluent.

Supplementary Note 2: Material sources.

The following reagents were purchased from commercial suppliers and used as received: *N*-bromosuccinimide (NBS, Wako Pure Chemical Industries), dichloromethane (superdehydrated, Wako Pure Chemical Industries), *n*-butyllithium (^{n}BuLi, 1.6 M in hexane, Kanto Chemical), tetrahydrofuran (THF, superdehydrated, stabilized with 2,6-di-*t*-butyl-*p*-cresol, Wako Pure Chemical Industries), dimethyl disulfide (Wako Pure Chemical Industries), 2,3,5,6-tetrafluoro-7,7,8,8-tetracyanoquinodimethane (**F$_4$**, Tokyo Chemical Industry), 2,5-difluoro-7,7,8,8-tetracyanoquinodimethane (**F$_2$**, Tokyo Chemical Industry), tetra-*n*-butylammonium tetrafluoroborate (^{n}Bu$_4$N•BF$_4$, Tokyo Chemical Industry), tetra-*n*-butylammonium hexafluorophosphate (^{n}Bu$_4$N•PF$_6$, Aldrich), carbon paste (XC-12, DO-TITE, Fujikura Kasei), and silver paste (D-500, DOTITE, Fujikura Kasei). 2,2'-Bi(3,4-ethylenedithiothiophene) was synthesized according to the literature.[2]

Supplementary Note 3: Synthesis of donor 2S and charge-transfer salt 2S•BF$_4$.

A donor **2S** was designed to have methylthio groups at its ends. These groups are small enough not interfere with the π-stacking of the molecule during crystallization, similar to **2O**.[1] Initially, we attempted to dimerize unsubstituted or dibrominated monomers, followed by lithiation and methylthio substitution.[1,3] However, these attempts resulted in low yields. Therefore, we modified the synthesis route by starting with bromination of unsubstituted dimer **1**, followed by lithiation and methyl thiolation, as shown in Fig. 3a and Methods section in the text.

A donor **2S** (4.3 mg, 10 μmol) was placed in one side of an H-shaped cell equipped with a glass filter, and ^{n}Bu$_4$N•BF$_4$ (2 × 10 mg) was placed in each side of the cell, respectively. The compounds were dissolved in acetonitrile (total 10 mL) under ultrasonic irradiation for >10 min. Two pre-annealed platinum electrodes were inserted into each side of the cell, and the cell was kept at 30 °C for 2 h. Then, a constant current of 0.2 μA was applied to the solution at the temperature for at least 2 d to afford black needle-like crystals of **2S**•BF$_4$ (typical size: ~300 × ~30 × ~30 μm^3; Fig. 3b and Supplementary Fig. 2). The crystal structure and

chemical composition were identified by the single-crystal X-ray structural analysis (Supplementary Table 1).

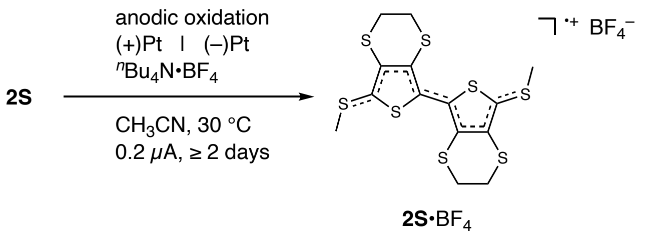

Supplementary Fig. 2: Synthesis of 2S•BF₄.

3. Electrochemical properties

Supplementary Note 4: Cyclic voltammetry measurements.

By using a silver-silver chloride electrode (Ag/AgCl/1 M KCl) as the reference electrode, we determined E_{ox1} and E_{ox2} values as the average of the potentials at the peak tops of the first and second oxidation and reduction processes[4] to be 0.622 and 0.951 V for **2O**, 0.805 and 1.05 V for **2S**, respectively (Table 1 and Supplementary Fig. 3). The HOMO levels were estimated from the first half-wave oxidation potentials by assuming the reference energy level of ferrocene/ferrocenium (Fc/Fc^+ = 0.486 V *vs*. Ag/AgCl/1 M KCl measured under the identical conditions) to be 4.8 eV from the vacuum levels,[5] to be −4.94 and −5.13 eV for **2O** and **2S**, respectively. Likewise, E_{red1} and E_{red2} of the first and second reduction processes were 0.652/0.024 V for **F₄**, 0.417/–0.202 V for **F₂**, respectively; the LUMO levels were determined to be −4.98 and −4.74 eV for **F₄** and **F₂**, respectively.

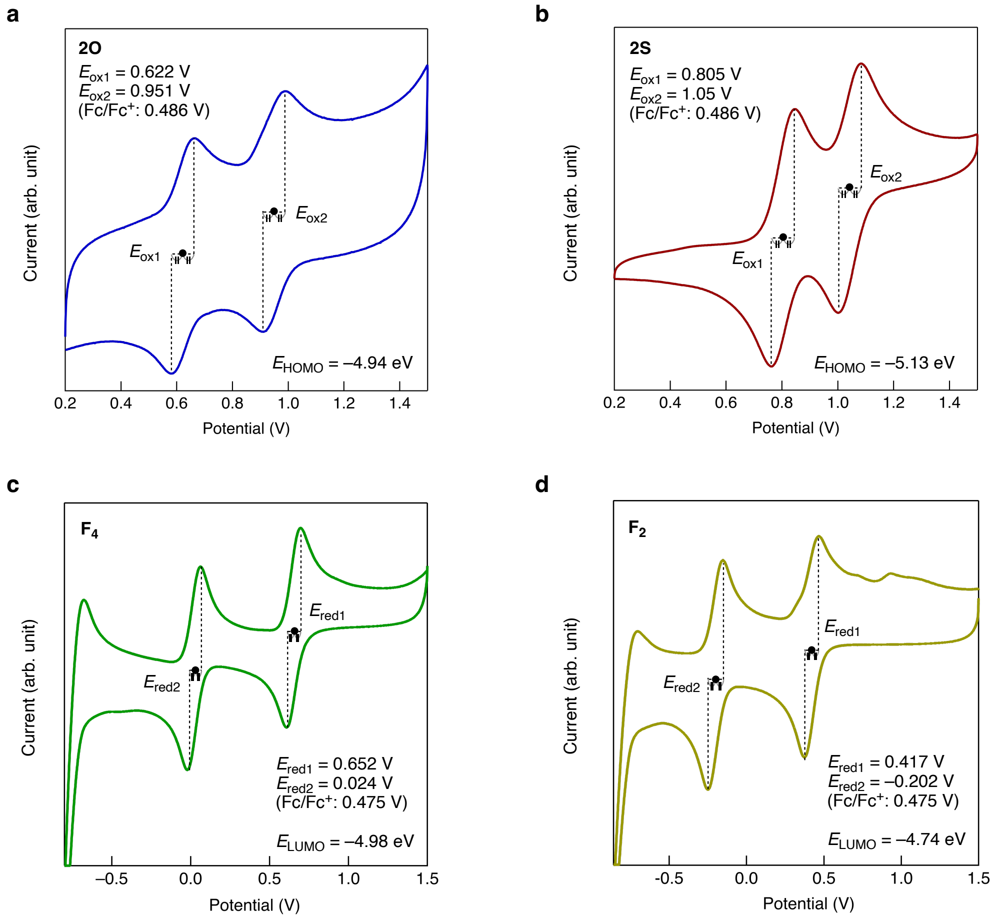

Supplementary Fig. 3: Cyclic voltammograms of donors and acceptors. a, **2O**. **b**, **2S**. **c**, $\mathbf{F_4}$. **d**, $\mathbf{F_2}$. The HOMO and LUMO levels were determined E_{ox1} and E_{red1}, respectively (Ag/AgCl/1 M KCl).

4. Theoretical calculations for isolated molecules

Supplementary Note 5: Calculation of molecular structures.

We calculated the energy levels and shapes of orbitals for donors and acceptors after structural optimization on the Gaussian09 program[6] at the density functional theory (DFT) level with the (unrestricted) B3LYP functional, the gradient correction of the exchange functional by Becke[7,8] and the correlation functional by Lee, Yang and Parr[9] and the 6-31G(d) split valence plus polarization basis set.[10,11,12] The Cartesian coordinates for the optimized geometries are shown in Supplementary Tables 6 and 8–12. The calculated shapes of HOMO for neutral **2S**, SOMO for one-electron-oxidized donors ($\mathbf{2O}^{\bullet+}$ and $\mathbf{2S}^{\bullet+}$), and SOMO for one-electron-reduced acceptors ($\mathbf{F_4}^{\bullet-}$ and $\mathbf{F_2}^{\bullet-}$) are visualized on GaussView 5.0[13] in Supplementary Figs. 4 and 5. The optimized structure of neutral **2S** showed a twisting angle between two thiophene rings (i.e., a

torsional angle $|\theta_1|$ (C^1-C^2-C^3-C^4) = 90.7° in Supplementary Fig. 4a; the calculated geometry was shown in Supplementary Table 6), mainly due to the steric repulsion between close two S atoms,[14] while that of **2O** is nearly planar, as observed in the single-crystal structure.[1] In contrast, the most stable conformer of **2S**$^{•+}$ was predicted to be planar (calculated $|\theta_1|$ = 161° in Fig 4b and Supplementary Fig. 5b), like **2O**$^{•+}$ [1] (calculated $|\theta_1|$ = 180° in Supplementary Fig. 5a). These planar structures of donors in the oxidized states are appropriate for π-stacking with planar acceptor molecules $\mathbf{F_4}$ and $\mathbf{F_2}$ in the reduced states (Fig. 4b and Supplementary Fig. 5c,d) for the formation of mixed-stack complexes. To ignore the contribution of the twisting and compare the energy levels of the neutral donor and acceptor before the complexation, the orbital energy levels of the planarized **2S** were calculated using a transient structure with the torsional angle $|\theta_1|$ to be 180°; the calculated geometry was shown in Supplementary Table 7. The calculated energy levels of neutral **2O**, **2S** with $|\theta_1|$ of 180°, $\mathbf{F_4}$, and $\mathbf{F_2}$ are shown in Fig. 4a and Supplementary Fig. 6. On the other hand, these calculations indicate that the C–S bonds between the C atoms at the 5-position of thiophene and the S atoms of the terminal MeS group of neutral **2O**[1] and **2S** oriented orthogonal to the π-plane (i.e., calculated $|\theta_2|$ of **2S** = 87.3° in Supplementary Fig. 4a); while the bonds in **2O**$^{•+}$ [1] and **2S**$^{•+}$ are coplanar to the π-planes (calculated $|\theta_2|$ = 180°; Supplementary Fig. 5a,b), as observed in the single-crystal of the charge-transfer salt **2O**•BF_4[1] and **2S**•BF_4 (observed $|\theta_2|$ = 178.7(3)° in Supplementary Fig. 8). The average periodicity of horizontally nodal orbitals[15] of **2O**$^{•+}$, **2S**$^{•+}$, $\mathbf{F_4}^{•-}$, and $\mathbf{F_2}^{•-}$ were calculated using the S–S distances of methylthio groups for **2O**$^{•+}$ and **2S**$^{•+}$ and N–N distances of the cyano groups for $\mathbf{F_4}^{•-}$ for $\mathbf{F_2}^{•-}$ by dividing the number of the nodes.

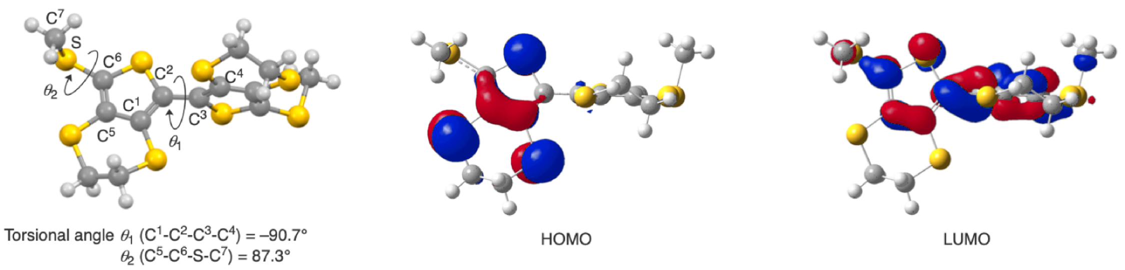

Supplementary Fig. 4: Geometry and molecular orbitals of neutral 2S. a, The optimized structure shown with the torsional angles θ_1 (C^1-C^2-C^3-C^4) and θ_2 (C^5-C^6-S-C^7). **b**, Calculated HOMO shape. **c**,

Calculated LUMO shape. Atoms were colored as follows; yellow: sulfur; gray: carbon; white: hydrogen.

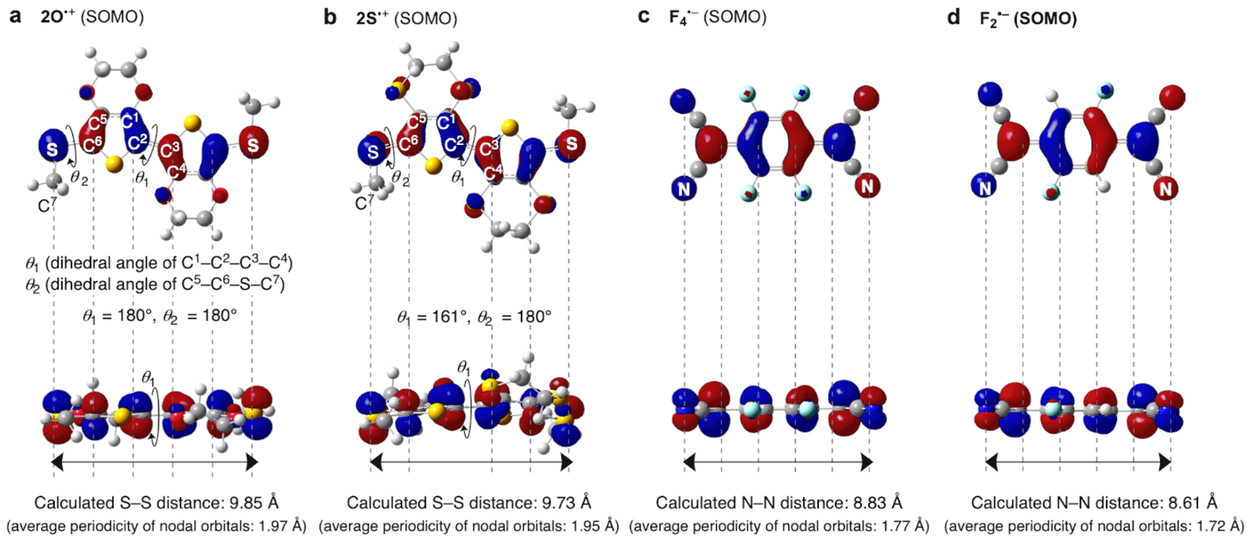

Supplementary Fig. 5: Calculated SOMO shapes of donors in a radical cation form and acceptors in a radical anion form. a, Radical cation **2O**$^{\bullet+}$.[1] **b**, Radical cation **2S**$^{\bullet+}$. **c**, Radical anion **F$_4$**$^{\bullet-}$. **d**, Radical anion **F$_2$**$^{\bullet-}$. Atoms were colored as follows; red: oxygen; yellow: sulfur; gray: carbon; light green: fluorine; white: hydrogen. The calculated S–S and N–N distances and the average periodicity of horizontally nodal orbitals are shown.

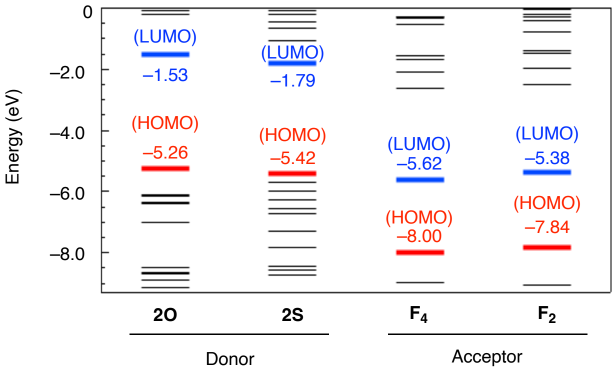

Supplementary Fig. 6: Calculated energy levels of orbitals for neutral donors and acceptors. In the calculation of **2S**, the dihedral angle $|\theta_1|$ between two planes was constrained at 180°. The levels of HOMO of **2O** and **2S** donors and LUMO of **F$_4$** and **F$_2$** acceptors were comparable, enhancing their hybridization.

5. Single-crystal structures

Supplementary Note 6: Single-crystal XRD measurements.

The in-house single-crystal X-ray diffractiometer (XRD) analyses of a neutral donor **2S** (Supplementary Fig. 7 and Supplementary Table 1) and a charge-transfer salt **2S•**BF_4 (Supplementary Fig. 8 and Supplementary Table 1) were performed using a Rigaku MercuryII CCD X-ray diffractometer (Mo K_{α}, X-ray wavelength λ = 0.71073 Å). The structures were analyzed by a direct method (SHELXT[16] version 2018/2) and refined with full-matrix least-squares technique (SHELXL version 2018/3) using an Olex2[17]-1.2 (OlexSys) software. Anisotropic thermal parameters were applied to all non-hydrogen atoms. The hydrogen atoms were generated geometrically. The single-crystal structure of neutral **2S** consists of crystallographically-independent half molecules that possess disordered ethylene groups **2S** with the occupancy of 72:28 (Supplementary Fig. 7). The single-crystal structure of charge-transfer salt **2S•**BF_4 consists of crystallographically-independent half molecules that possess disordered ethylene groups **2S** with the occupancy of 67:33 and fluorine atoms with the occupancy of 52:48 (Supplementary Fig. 8).

The synchrotron single-crystal XRD analyses of mixed-stack complexes at 300 K (Supplementary Figs. 9–12, 14, and Supplementary Table 2) and **2S–**$\mathbf{F_4}$ at 200 K (Supplementary Figs. 13 and 15) were performed and on BL02B1 (X-ray wavelength λ = 0.30960 Å) at a synchrotron facility SPring-8 in Japan.[18] A N_2-gas-blowing device was employed for the low-temperature measurements. A two-dimensional detector CdTe PILATUS was used to record the diffraction pattern. The intensities of Bragg reflections were collected by CrysAlisPro program.[19] Intensities of equivalent reflections were averaged, and the structural parameters were refined by using Jana2006.[20] Fluorine atoms of **2O–**$\mathbf{F_2}$ and **2S–**$\mathbf{F_2}$ were positionally disordered with the occupancy of 94:6 and 86:14 (Supplementary Fig. 9b,d). The bond lengths analyses are shown in Fig. 5. In the synchrotron radiation experiments at 300 K, we observed significant X-ray diffuse scattering along the c^*-axis and b^*+c^*-direction (Supplementary Fig. 14). This scattering can be induced by the π-dimerization fluctuation between donors and acceptors along the a-axis (i.e., the π-stacking direction) in columns, possibly resulting in the intercolumnar interactions to fluctuate the molecular arrangement along

the c-axis. The scattering may be a precursor phenomenon for the one-dimensional nature as identified for TTF–CA.[21] Upon cooling to 200 K, the X-ray diffuse scattering disappeared (Supplementary Figs. 15 and 16), possibly due to the π-dimerization. Considering the magnetic characteristics that showed paramagnetic to non-magnetic transition at 282 K, this disappearance may be related to spin-Peierls-like π-dimerization, similar to those observed in one-dimensional charge-transfer complexes.[22] The disappearance supports the X-ray diffuse scattering occurring at 300 K is a precursor phenomenon prior to π-dimerization. The XRD at 200 K showed a superlattice with dimensions of $a \times 2b \times 2c$ (Supplementary Fig. 13). The superlattice peaks are observed only at $k + l = 2n$ in $a \times 2b \times 2c$ lattice. These peaks correspond to the reflection conditions of the A-base center. The observation enabled us to propose a possible dimer model for the molecular arrangement, as shown in Supplementary Fig. 17. The models suggest a stripe pattern of the π-dimerization, which should belong to a space group of $P1$ without an inversion center.

Supplementary Table 1: Crystallographic data for single-crystal 2S and 2S•BF$_4$.

Compounds	**2S**	**2S**•BF$_4$
Temperature / K	293	293
Formula	$C_{14}H_{14}S_8$	$C_{14}H_{14}S_8BF_4$
Formula weight	438.73	525.54
Crystal system	*monoclinic*	*monoclinic*
Space group	$P2_1/n$ (#14)	$C2/c$ (#15)
a / Å	8.1995(4)	15.416(3)
b / Å	13.2416(8)	21.8312(15)
c / Å	8.6626(5)	7.6027(13)
α / deg.	90	90
β / deg.	109.929(6)	127.78(3)
γ / deg.	90	90
V / Å^3	884.21(9)	2022.4(8)
Z	2	4
D_{calc} / g cm^{-3}	1.648	1.726
$R_{\rm int}$	0.0165	0.0330
R_1 ($I > 2.00\sigma(I)$)	0.0241	0.0387
wR_2 (all reflections)	0.0656	0.0950
GOF	1.040	1.029
CCDC	2264341	2264342

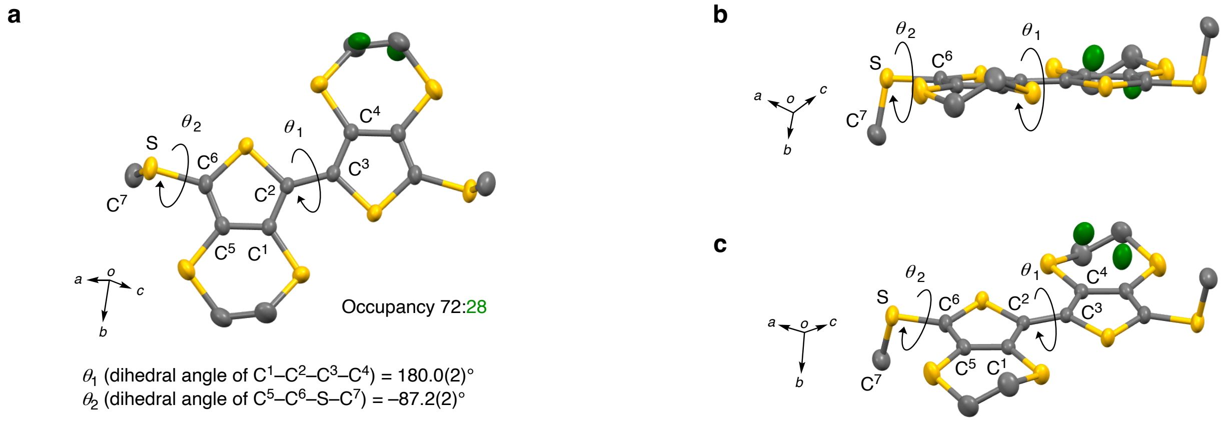

Supplementary Fig. 7: Single-crystal structure of 2S at 293 K. The structure is displayed in ORTEP (50% thermal ellipsoid) drawing along the π-stacking direction (**a**), from perpendicular direction to the π-plane of the molecule (**b**), and along the slanting direction to the π-plane (**c**). Disordered carbon atoms in the minor occupancy were colored in green. Other carbon and sulfur atoms were colored in gray and yellow, respectively. Hydrogens were omitted for clarity.

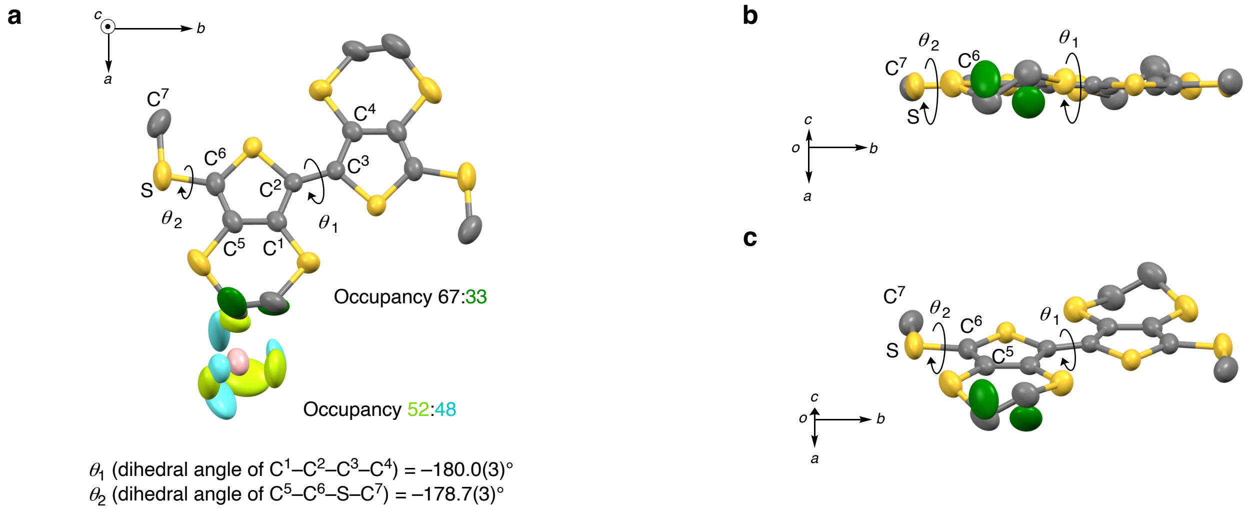

Supplementary Fig. 8: Single-crystal structure of 2S•BF_4 at 293 K. The structure is displayed in ORTEP (50% thermal ellipsoid) drawing along the π-stacking direction (**a**), from perpendicular direction to the π-plane of the molecule (**b**), and along the slanting direction to the π-plane (**c**). Disordered carbon and fluorine atoms in the minor occupancy were colored in green and aqua, respectively. Other carbon, fluorine, sulfur, and boron atoms were colored in gray, light green, yellow, and orange, respectively. Hydrogens and anions were omitted for clarity (**b**,**c**).

Supplementary Table 2: Crystallographic data for single-crystal mixed-stack complexes.

Compounds	**2O–F$_4$**	**2O–F$_2$**	**2S–F$_4$**	**2S–F$_2$**
Temperature / K	300	300	300	300
Formula	$C_{26}H_{14}F_4N_4O_4S_4$	$C_{26}H_{16}F_2N_4O_4S_4$	$C_{26}H_{14}F_4N_4S_8$	$C_{26}H_{16}F_2N_4S_8$
Formula weight	650.65	614.67	714.89	676.91
Crystal system	*monoclinic*	*monoclinic*	*monoclinic*	*monoclinic*
Space group	$P2_1/n$ (#14)	$P2_1/n$ (#14)	$P2_1/n$ (#14)	$P2_1/n$ (#14)
a / Å	6.7240(2)	6.6638(9)	6.8223(2)	6.8082(2)
b / Å	20.9067(7)	20.668(3)	21.9312(7)	22.0498(7)
c / Å	9.7855(3)	9.5765(13)	9.9232(3)	9.9559(3)
α / deg.	90	90	90	90
β / deg.	105.654(8)	104.565(7)	107.541(8)	107.967(8)
γ / deg.	90	90	90	90
V / Å^3	1324.59(9)	1276.5(3)	1415.68(10)	1421.69(10)
Z	2	2	2	2
D_{calc} / g cm^{-3}	1.9529	2.0264	1.677	1.67
R_{int}	0.0572	0.1011	0.0658	0.0646
R_1 ($I > 2.00\sigma(I)$)	0.0513	0.0503	0.0343	0.0394
wR_2 (all reflections)	0.0677	0.0742	0.0554	0.0604
GOF	2.47	1.98	2.05	2.00
CCDC	2264325	2264326	2264327	2264331

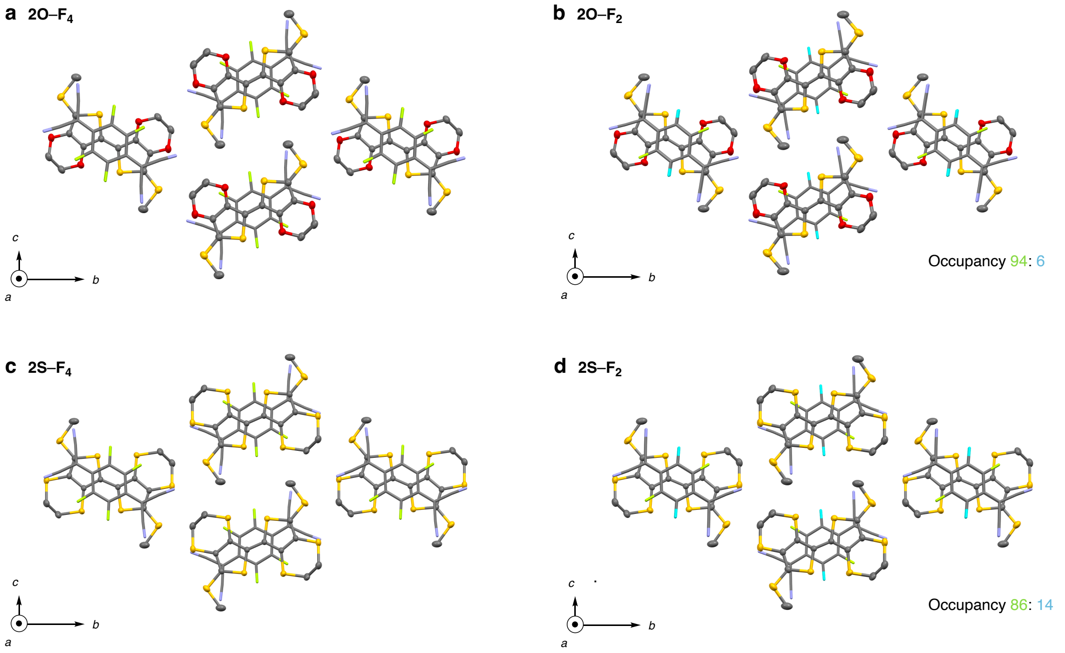

Supplementary Fig. 9: Single-crystal structures of mixed-stack complexes at 300 K. a, **2O–F$_4$**. **b**, **2O–F$_2$**. **c**, **2S–F$_4$**. **d**, **2S–F$_2$**. Locationally disordered fluorine atoms in **2O–F$_2$** and **2S–F$_2$** were colored in aqua (**d**). Other atoms were colored as follows; yellow: sulfur; red: oxygen; gray: carbon; blue: nitrogen; light green: fluorine. Hydrogens were omitted for clarity. ORTEP (50% thermal ellipsoid) and wire drawing for donor and acceptor, respectively.

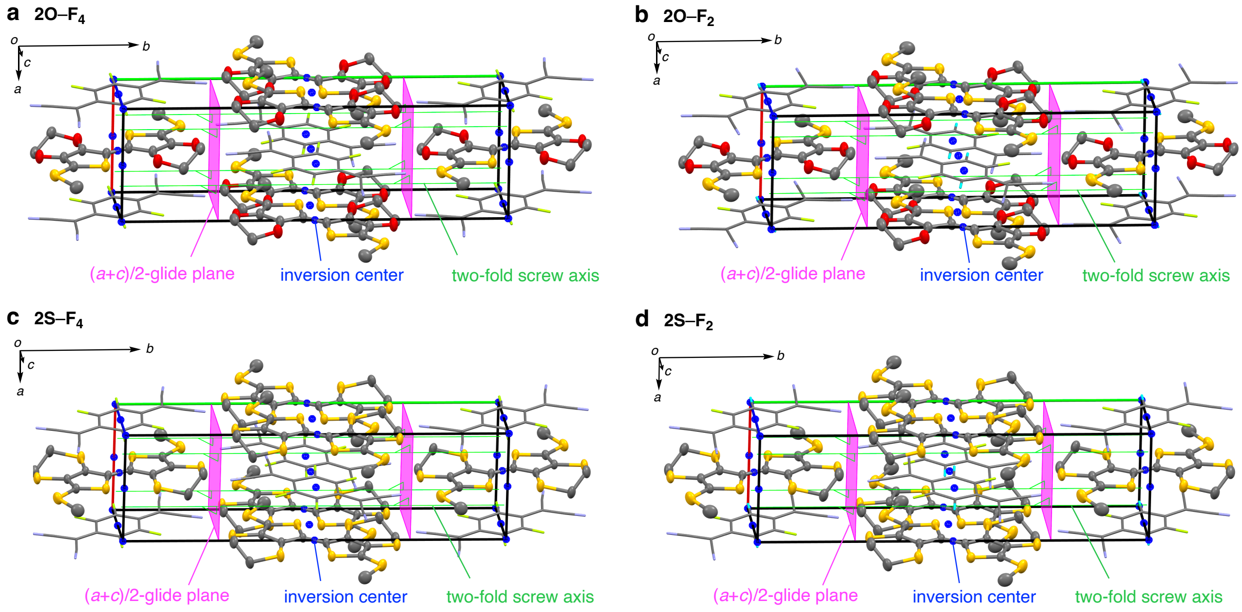

Supplementary Fig. 10: Symmetry elements in the single-crystal structure of mixed-stack complexes at 300 K. a, **2O–F$_4$**. **b**, **2O–F$_2$** (major occupancy). **c**, **2S–F$_4$**. **d**, **2S–F$_2$** (major occupancy). The structures are displayed in ORTEP (donors, 50% thermal ellipsoid) and wire drawing for donor and acceptor, respectively. Hydrogens were omitted for clarity. Atoms were colored as follows; yellow: sulfur; red:

oxygen; gray: carbon; blue: nitrogen; light green: fluorine. Disordered atoms and hydrogens were omitted for clarity.

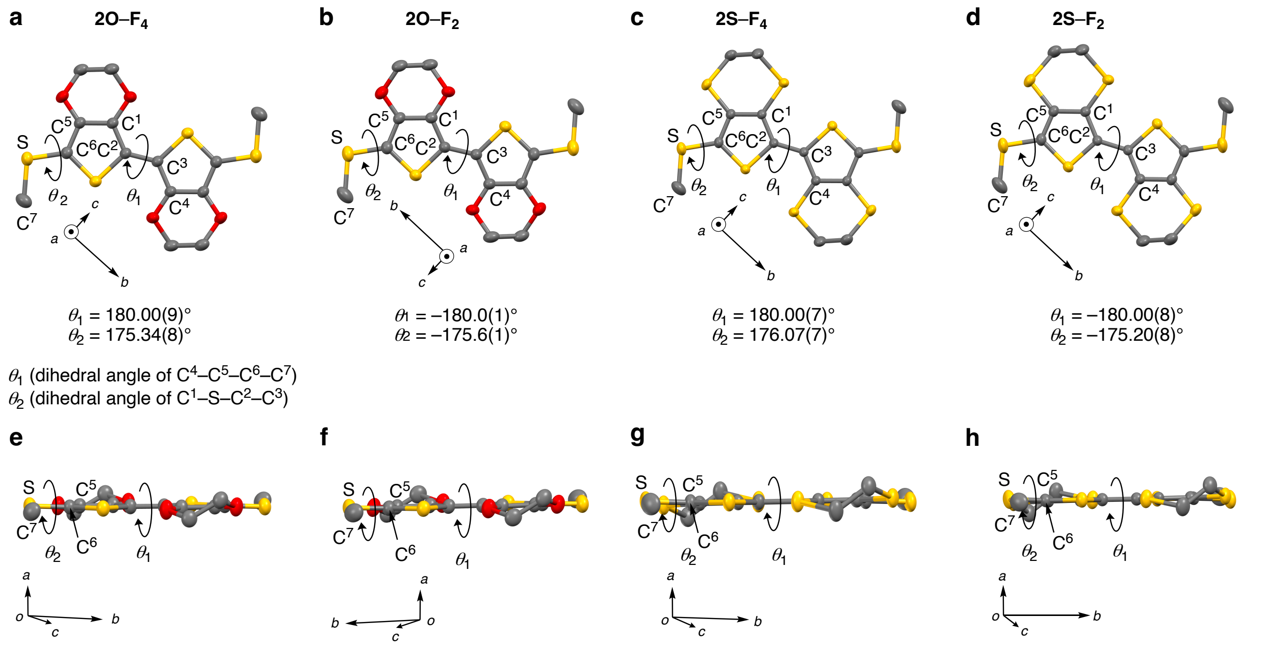

Supplementary Fig. 11: Single-crystal structures of donors in mixed-stack complexes at 300 K. Structures of **2O–F₄** (**a**,**e**), **2O–F₂** (**b**,**f**), **2S–F₄** (**c**,**g**), and **2S–F₂** (**d**,**h**) are shown along the *a*-axis (**a**,**b**,**c**,**d**) and direction perpendicular to the π-stacking (**e**,**f**,**g**,**h**) displayed in ORTEP (50% thermal ellipsoid) drawing. Atoms were colored as follows; yellow: sulfur; red: oxygen atom; gray: carbon atom. Hydrogens were omitted for clarity.

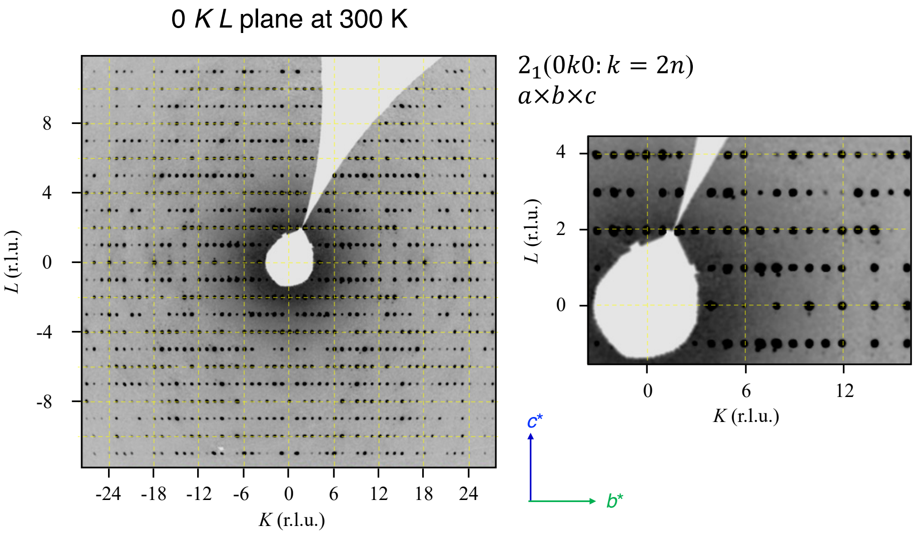

Supplementary Fig. 12: Single-crystal X-ray diffraction precession image of the 0 *K* *L* plane in the

reciprocal lattice of 2S–F_4 at 300 K.

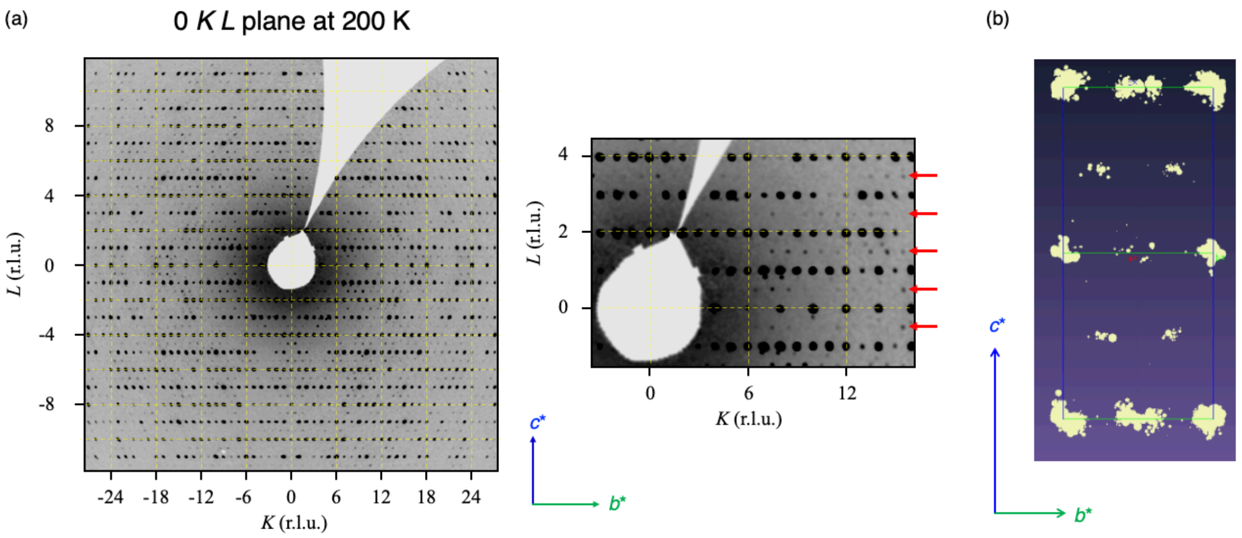

Supplementary Fig. 13: Single-crystal X-ray diffraction precession image of the 0 *K L* plane of 2S–F_4 at 200 K. (a) The reciprocal lattice. (b) The projection of the reciprocal lattice points to the b^*c^* plane ($-1 \leq k, l \leq 1$, b). Superlattice peaks are observed only at $k + l = 2n$ in the in $a \times 2b \times 2c$ lattice, which corresponds to reflection conditions of the A-base center.

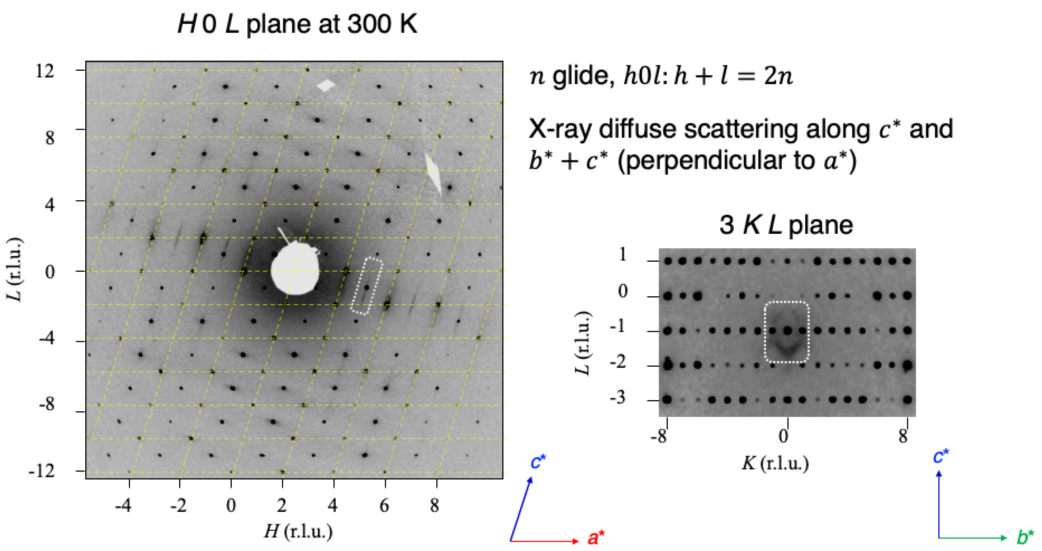

Supplementary Fig. 14: Single-crystal X-ray diffraction precession images of the *H* 0 *L* (a) and 3 *K L* (b) planes in the reciprocal lattice of 2S–F_4 at 300 K. The X-ray diffuse scattering extends along the c^*-axis and b^*+c^*-direction.

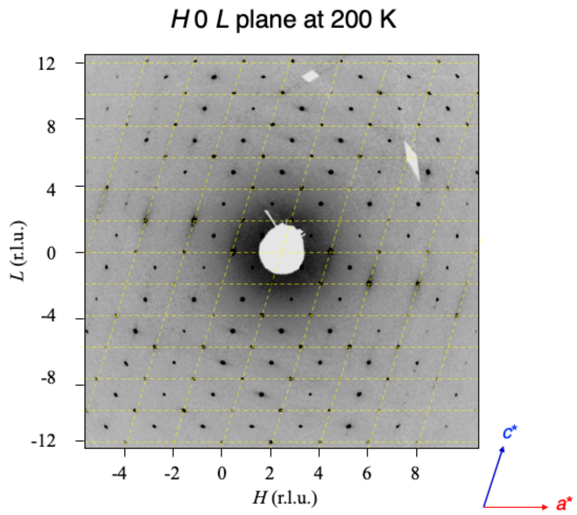

Supplementary Fig. 15: Single-crystal X-ray diffraction precession image of the *H* 0 *L* plane in the reciprocal lattice of 2S–F_4 at 200 K. Significant X-ray diffuse scattering was not detected.

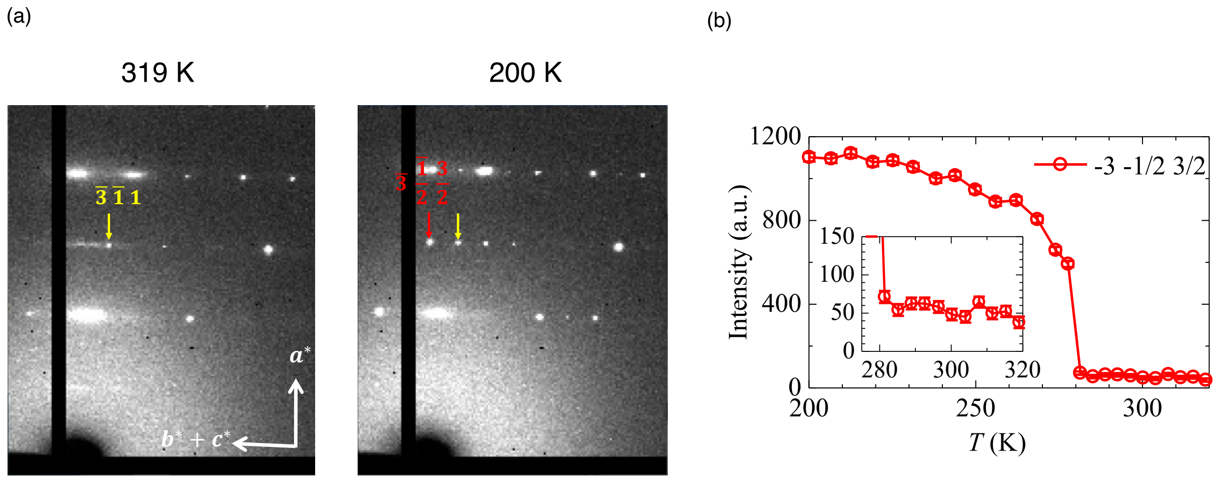

Supplementary Fig. 16: Temperature-dependence of single-crystal X-ray diffraction precession of **2S–F_4.** (a) Precession images at 319 K and 200 K. (b) Temperature-dependent intensity of the signals for (–3,–1/2,3/2). The X-ray diffuse scattering along the b^*+c^*-direction disappeared and the superlattice peaks at $k + l = 2n$ appeared at approximately 282 K.

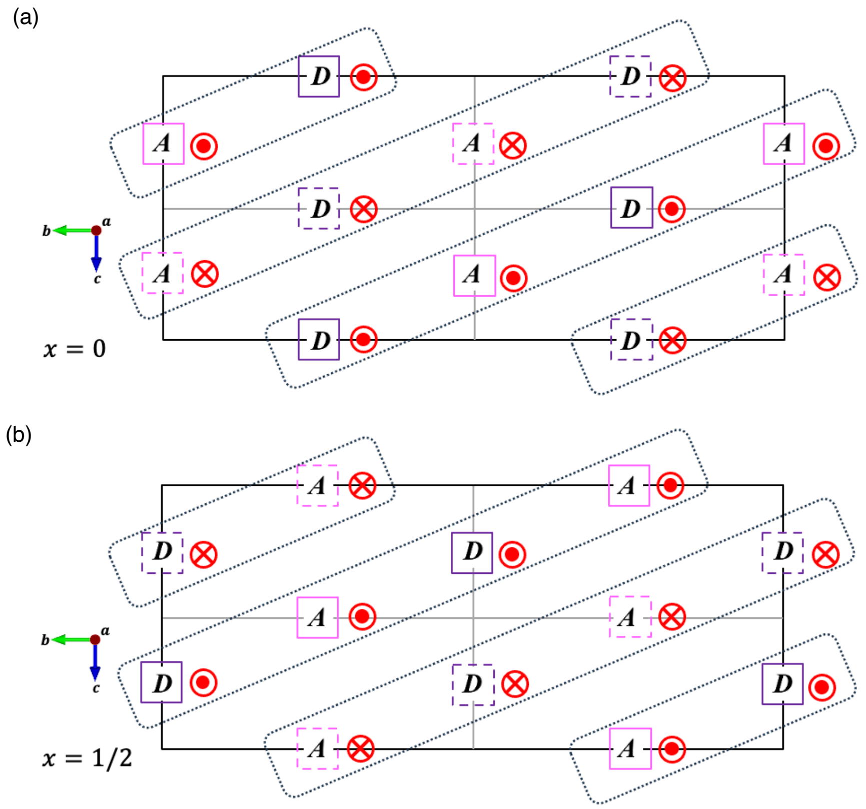

Supplementary Fig. 17: Possible Models proposed for the molecular arrangement of 2S–F_4 superlattice observed at 200 K. a, *x* = 0. **b**, *x* = 1/2. The **A/D** molecules squared in the solid and dashed lines move in the opposite direction along the *a*-axis, leading to a stripe-pattern charge-densitgy wave arrangement in the *bc* plane. The model should belong to the space group of *P*1.

6. Theoretical calculations.

Supplementary Note 7: Band and crystal orbital calculations

Mixed-stack complexes, particularly those at the N–I boundary, may exhibit fluctuation in π-dimerization at room temperatures before undergoing structural changes. This was suggested by X-ray diffuse scattering that disappeared when cooled to 200 K, as well as EMV coupling (which will be discussed later). As have been analyzed for one-dimensional charge-transfer complexes display uniformly π-stacked single-crystal structures with dynamic fluctuations prior to the spin-Peierls-like π-dimerization,[22] we performed theoretical calculations based on the average single-crystal XRD structures. In the calculations, the locational disordering of **2O–F_2** and **2S–F_2** (Supplementary Fig. 9b,d) may have impacts on the electronic

structures and physical properties. To get an insight into the possible impacts, we performed the calculations not only for the major but also for the minor occupancies. The calculation conditions are shown in Methods section of the text. The results showed that complexes consistently exhibited half-filled 1D electronic structures (Fig. 4c and Supplementary Fig. 18). The real parts of the HOCO and lowest-unoccupied crystal orbitals (LUCO) at the Γ point (0, 0, 0) were visualized by VESTA[23] (Fig. 4d and Supplementary Fig. 19). The transfer integrals between a donor and the six neighboring molecules (donors and acceptors) were calculated (Supplementary Fig. 27 and Supplementary Table 3) to confirm the dominant intracolumnar interactions ($\boldsymbol{t_1}$ and $\boldsymbol{t_4}$).

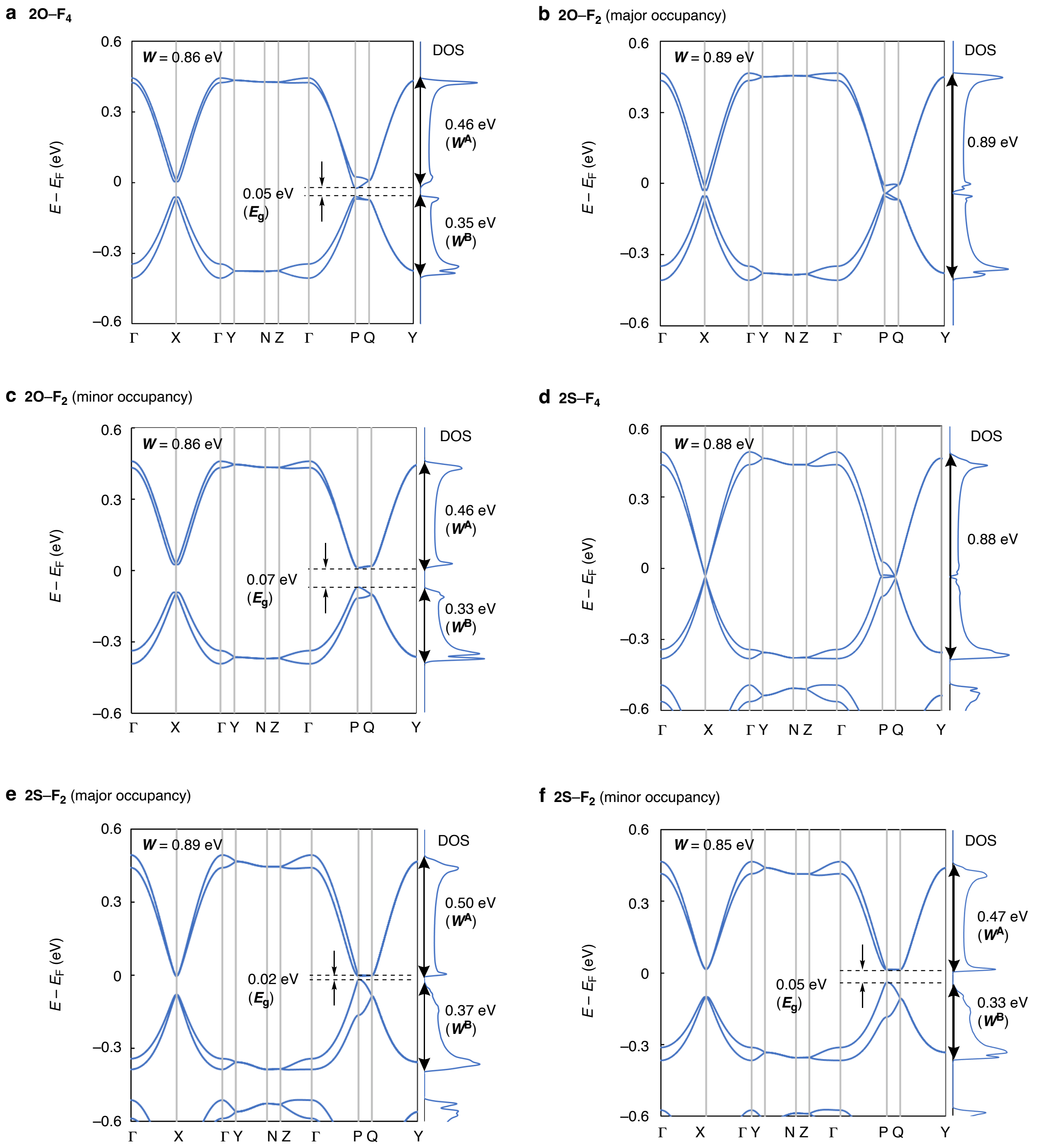

Supplementary Fig. 18: Band structures of mixed-stack complexes. **a**, **2O–F₄**. **b**, **2O–F₂** (major occupancy). **c**, **2O–F₂** (major occupancy). **d**, **2S–F₄**. **e**, **2S–F₂** (major occupancy). **f**, **2S–F₂** (minor occupancy). **Γ** (0, 0, 0), **X** (0.5, 0, 0), **Y** (0, 0.5, 0), **N** (0, 0.5, 0.5), **Z** (0, 0, 0.5), **P** (–0.5, 0, 0.5), **Q** (–0.5, 0.5, 0.5). The Fermi levels (E_F) are determined by occupying electrons according to the Fermi distribution function.

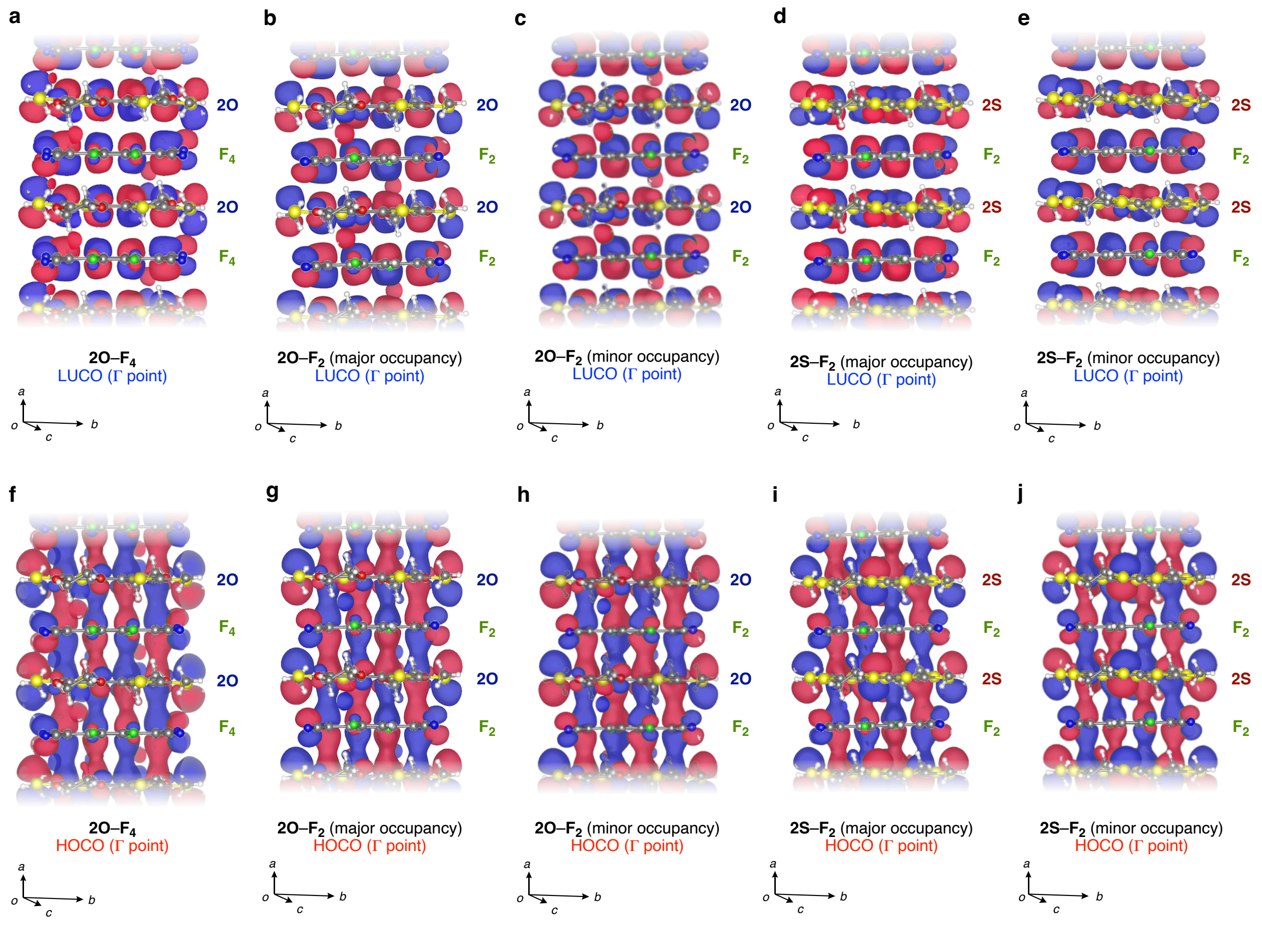

Supplementary Fig. 19: Crystal orbitals of mixed-stack complexes. a,**b**,**c**,**d**,**e**, The LUCO shapes of **2O–F$_4$** (**a**), **2O–F$_2$** (**b**, major occupancy), **2O–F$_2$** (**c**, minor occupancy), **2S–F$_2$** (**d**, major occupancy), and **2S–F$_2$** (**e**, minor occupancy). **f**,**g**,**h**,**i**,**j**, The HOCO shapes of **2O–F$_4$** (**f**), **2O–F$_2$** (**g**, major occupancy), **2O–F$_2$** (**h**, minor occupancy), **2S–F$_2$** (**i**, major occupancy), and **2S–F$_2$** (**j**, minor occupancy). Crystal orbitals were visualized by VESTA.[25] The orbitals with positive and negative phases were colored with magenta and navy, respectively. Atoms were colored as follows; yellow: sulfur; red: oxygen; gray: carbon; blue: nitrogen; light green: fluorine.

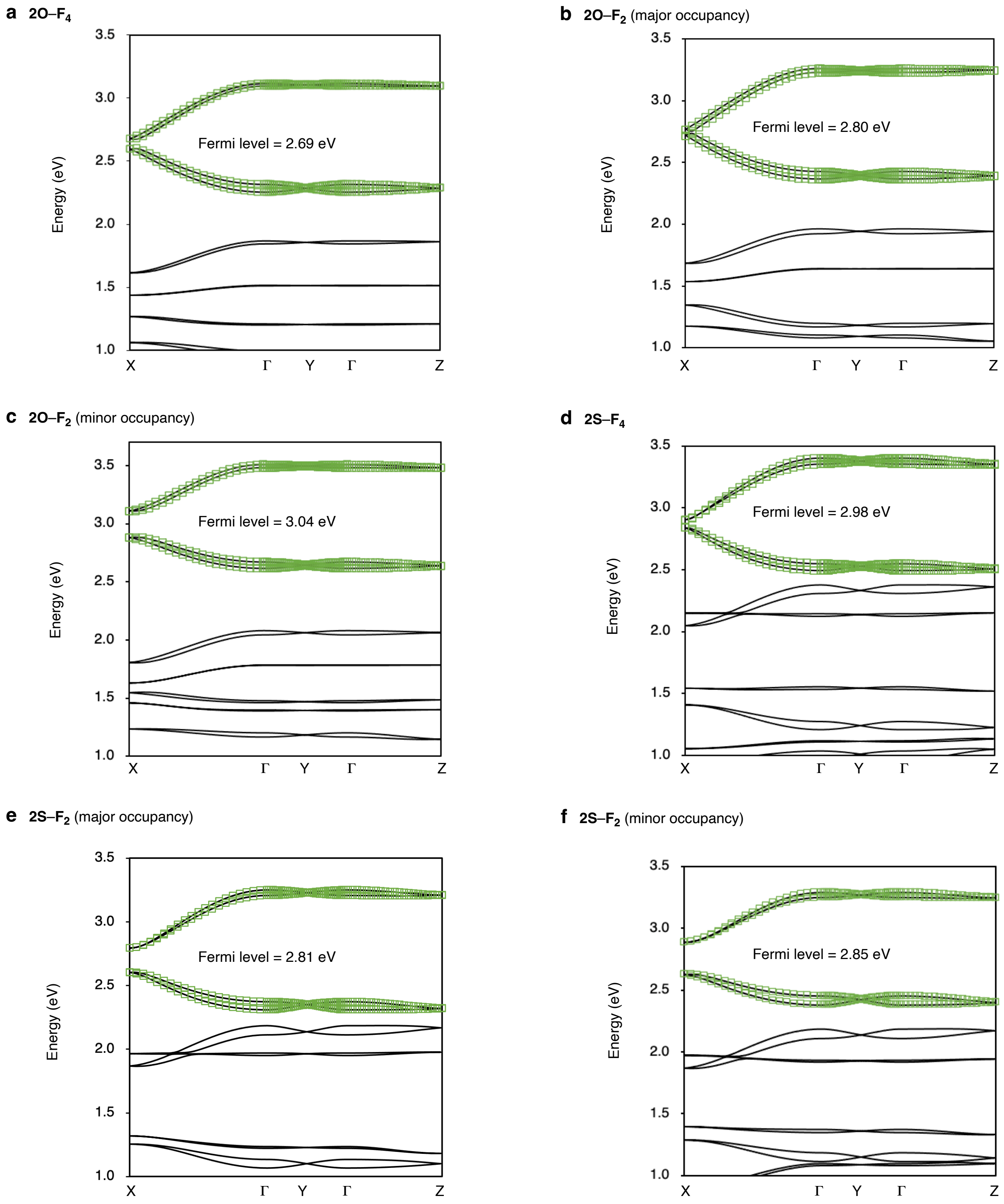

Supplementary Fig. 20: Wannier interpolation bands (shown in green squares) and band dispersion (shown in black solid lines). a, **2O–F_4**. **b**, **2O–F_2** (major occupancy). **c**, **2O–F_2** (minor occupancy). **d**, **2S–F_4**. **e**, **2S–F_2** (major occupancy). **f**, **2S–F_2** (minor occupancy).

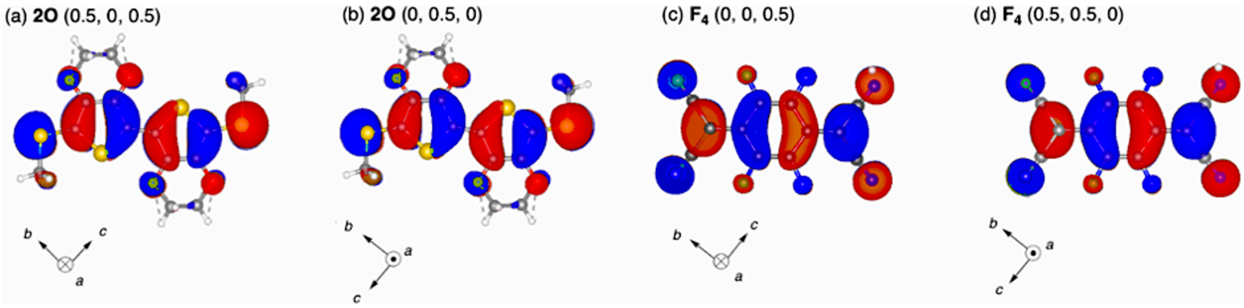

Supplementary Fig. 21: The maximally localized Wannier function of 2O–F_4 in a cell. Atoms surrounding the molecules were omitted for clarity. Atoms were colored as follows; yellow: sulfur; red: oxygen; gray: carbon; blue: nitrogen; light green: fluorine. **a**, **2O** (0.5, 0, 0.5). **b**, **2O** (0, 0.5, 0). **c**, $\mathbf{F_4}$ (0, 0, 0.5). **d**, $\mathbf{F_4}$ (0.5, 0.5, 0).

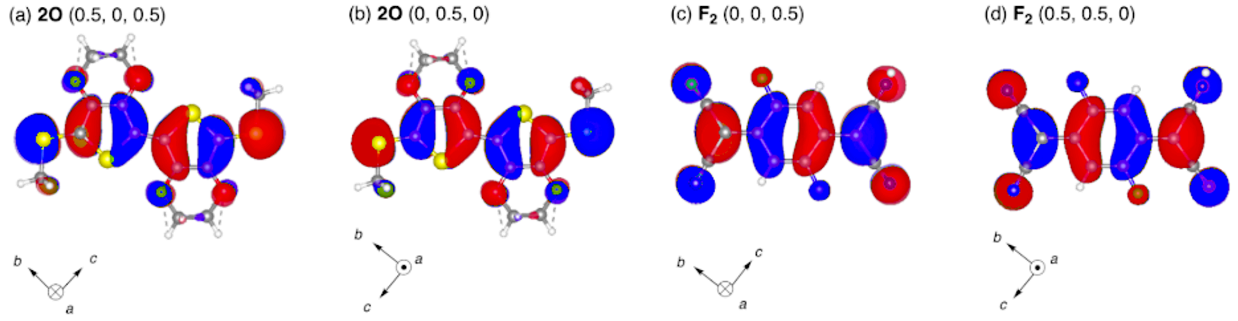

Supplementary Fig. 22: The maximally localized Wannier function of 2O–F_2 (major occupancy) in a cell. Atoms surrounding the molecules were omitted for clarity. Atoms were colored as follows; yellow: sulfur; red: oxygen; gray: carbon; blue: nitrogen; light green: fluorine. **a**, **2O** (0.5, 0, 0.5). **b**, **2O** (0, 0.5, 0). **c**, $\mathbf{F_2}$ (0, 0, 0.5). **d**, $\mathbf{F_2}$ (0.5, 0.5, 0).

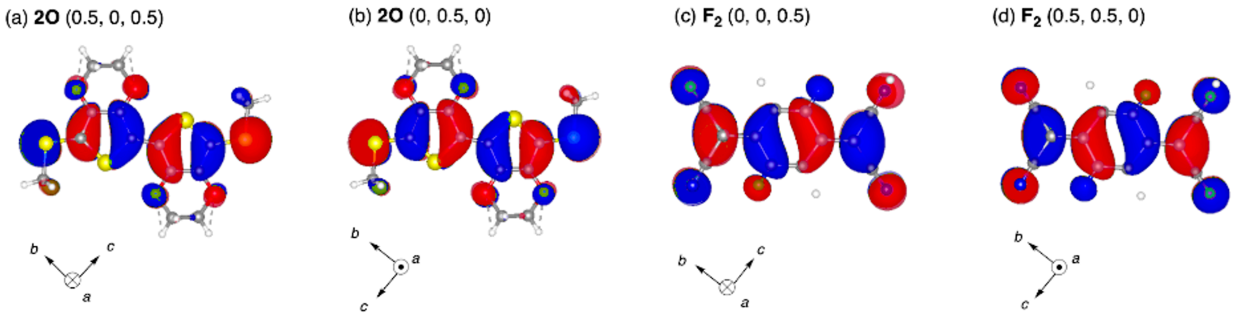

Supplementary Fig. 23: The maximally localized Wannier function of 2O–F_2 (minor occupancy) in a cell. Atoms surrounding the molecules were omitted for clarity. Atoms were colored as follows; yellow: sulfur; red: oxygen; gray: carbon; blue: nitrogen; light green: fluorine. **a**, **2O** (0.5, 0, 0.5). **b**, **2O** (0, 0.5, 0). **c**, $\mathbf{F_2}$ (0, 0, 0.5). **d**, $\mathbf{F_2}$ (0.5, 0.5, 0).

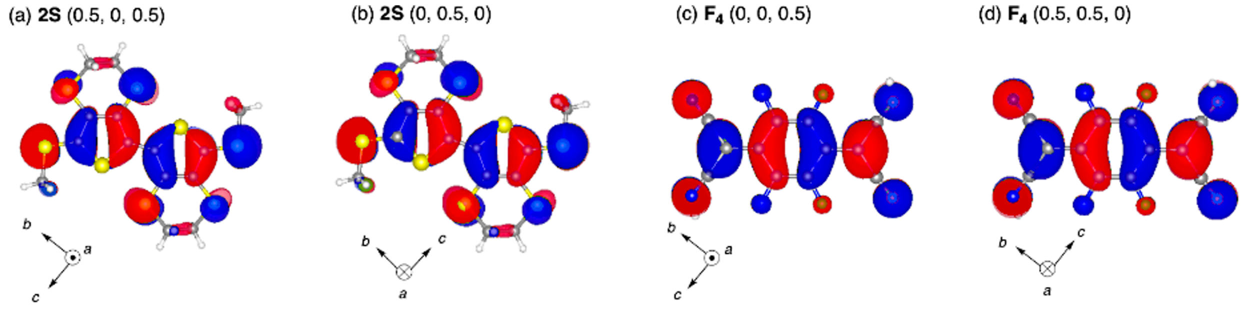

Supplementary Fig. 24: The maximally localized Wannier function of 2S–F_4 in a cell. Atoms surrounding the molecules were omitted for clarity. Atoms were colored as follows; yellow: sulfur; red: oxygen; gray: carbon; blue: nitrogen; light green: fluorine. **a**, **2S** (0.5, 0, 0.5). **b**, **2S** (0, 0.5, 0). **c**, **F_4** (0, 0, 0.5). **d**, **F_4** (0.5, 0.5, 0).

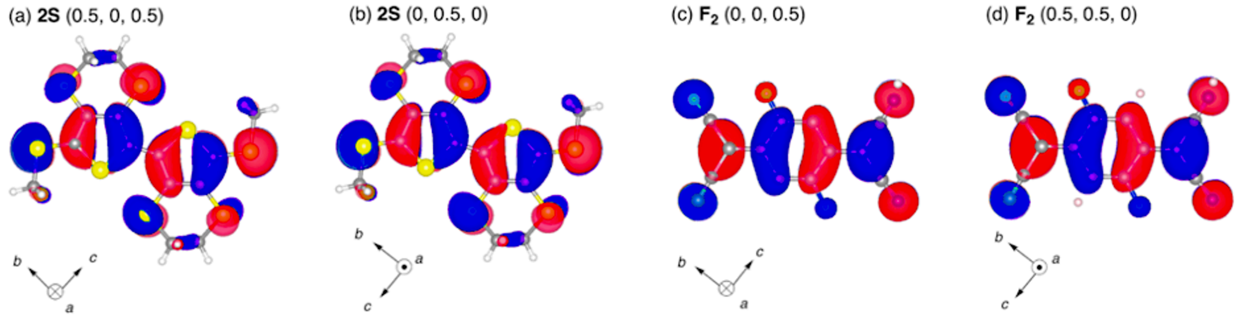

Supplementary Fig. 25: The maximally localized Wannier function of 2S–F_2 (major occupancy) in a cell. Atoms surrounding the molecules were omitted for clarity. Atoms were colored as follows; yellow: sulfur; red: oxygen; gray: carbon; blue: nitrogen; light green: fluorine. **a**, **2S** (0.5, 0, 0.5). **b**, **2S** (0, 0.5, 0). **c**, **F_2** (0, 0, 0.5). **d**, **F_2** (0.5, 0.5, 0).

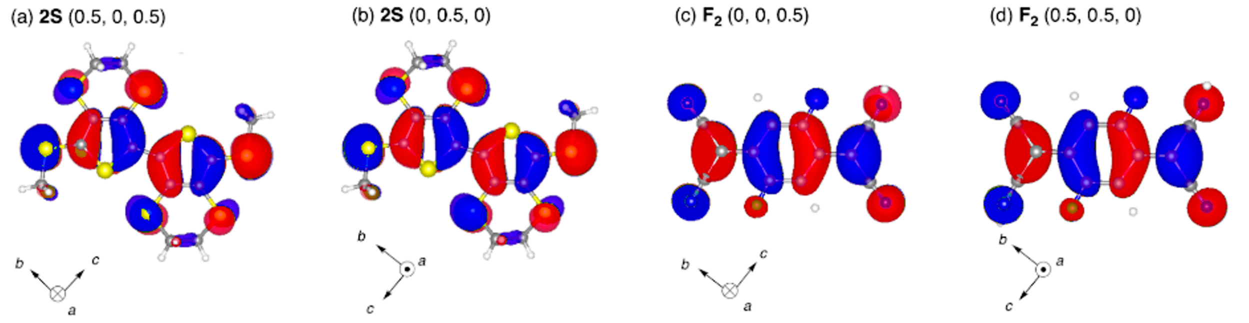

Supplementary Fig. 26: The maximally localized Wannier function of 2S–F_2 (minor occupancy) in a cell. Atoms surrounding the molecules were omitted for clarity. Atoms were colored as follows; yellow: sulfur; red: oxygen; gray: carbon; blue: nitrogen; light green: fluorine. **a**, **2S** (0.5, 0, 0.5). **b**, **2S** (0, 0.5, 0). **c**, **F_2** (0, 0, 0.5). **d**, **F_2** (0.5, 0.5, 0).

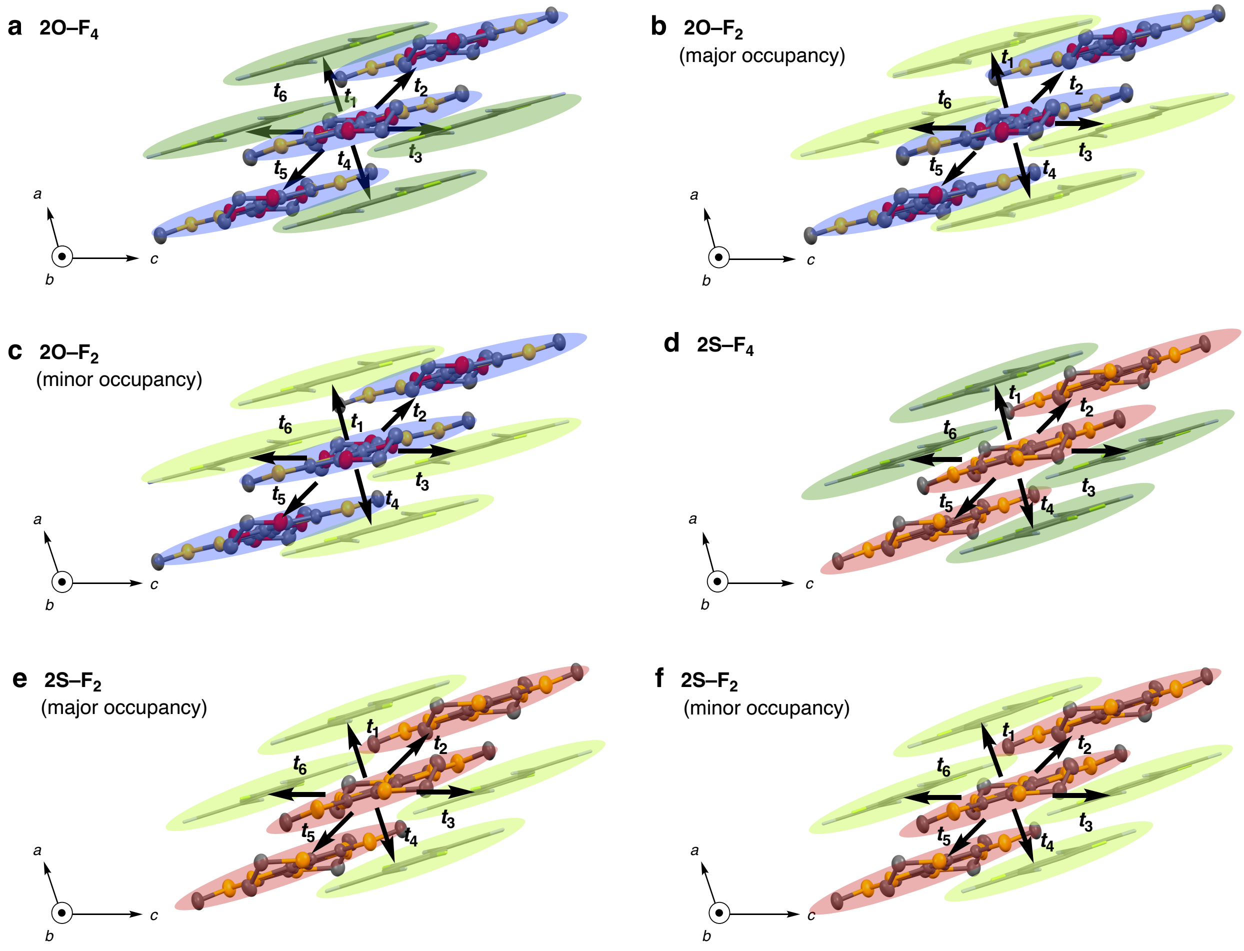

Supplementary Fig. 27: Labels for *t* values for mixed-stack complexes. a, **2O–F$_4$**. **b**, **2O–F$_2$** (major occupancy). **c**, **2O–F$_2$** (minor occupancy). **d**, **2S–F$_4$**. **e**, **2S–F$_2$** (major occupancy). **f**, **2S–F$_2$** (minor occupancy). The values were summarized in Supplementary Table 3. **2O**, **2S**, **F$_4$**, and **F$_2$** were colored with a blue, red, green, and light green background, respectively for clarity. Atoms were colored as follows; yellow: sulfur; red: oxygen; gray: carbon; blue: nitrogen; light green: fluorine.

Supplementary Table 3: Transfer integrals for mixed-stack complexes. The intracolumnar values ($\boldsymbol{t_1}$ and $\boldsymbol{t_4}$) are shown as $\boldsymbol{t_{DA}}$ in Table 1.

Complex	**2O–F$_4$**	**2O–F$_2$** (major occupancy)	**2O–F$_2$** (minor occupancy)	**2S–F$_4$**	**2S–F$_2$** (major occupancy)	**2S–F$_2$** (minor occupancy)
t_1 (eV)	0.203	0.209	0.197	0.208	0.206	0.187
t_2 (eV)	0.00497	−0.00429	−0.00395	0.0183	0.0187	0.0182
t_3 (eV)	0.00190	−0.00229	−0.00200	−0.000301	0.0005174	0.000759
t_4 (eV)	0.203	0.209	0.197	0.208	0.206	0.187
t_5 (eV)	0.00495	−0.00422	−0.00400	0.0183	0.0187	0.0182
t_6 (eV)	−0.0121	0.0141	0.0134	0.000953	0.00252	0.00190

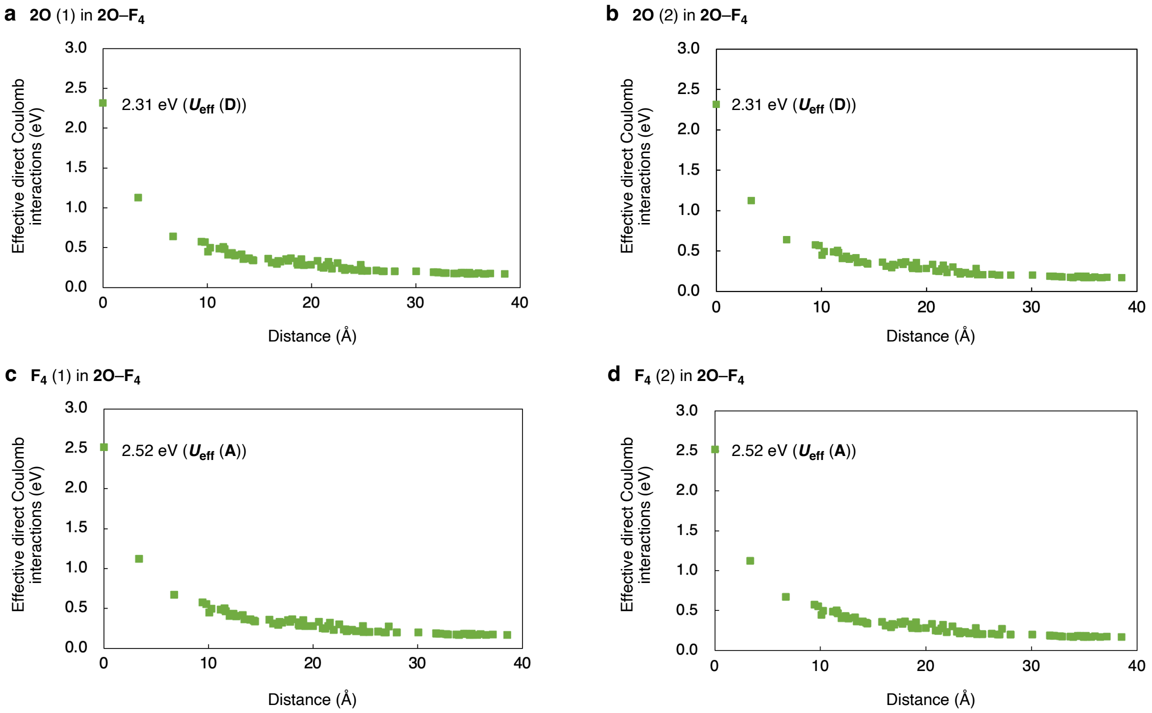

Supplementary Fig. 28: Effective direct Coulomb interactions in a cell of 2O–F$_4$. Values of donors (**a**,**b**), and acceptors (**c**,**d**).

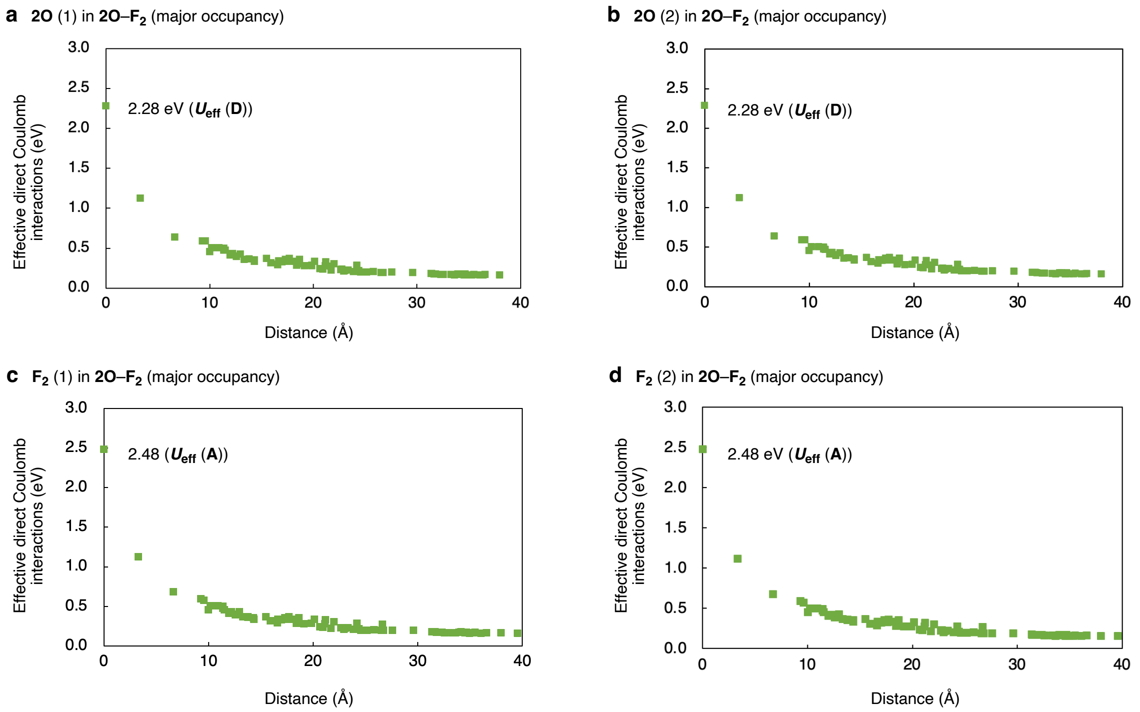

Supplementary Fig. 29: Effective direct Coulomb interactions in a cell of 2O–F_2 in the major occupancy. Values of donors (**a**,**b**), and acceptors (**c**,**d**).

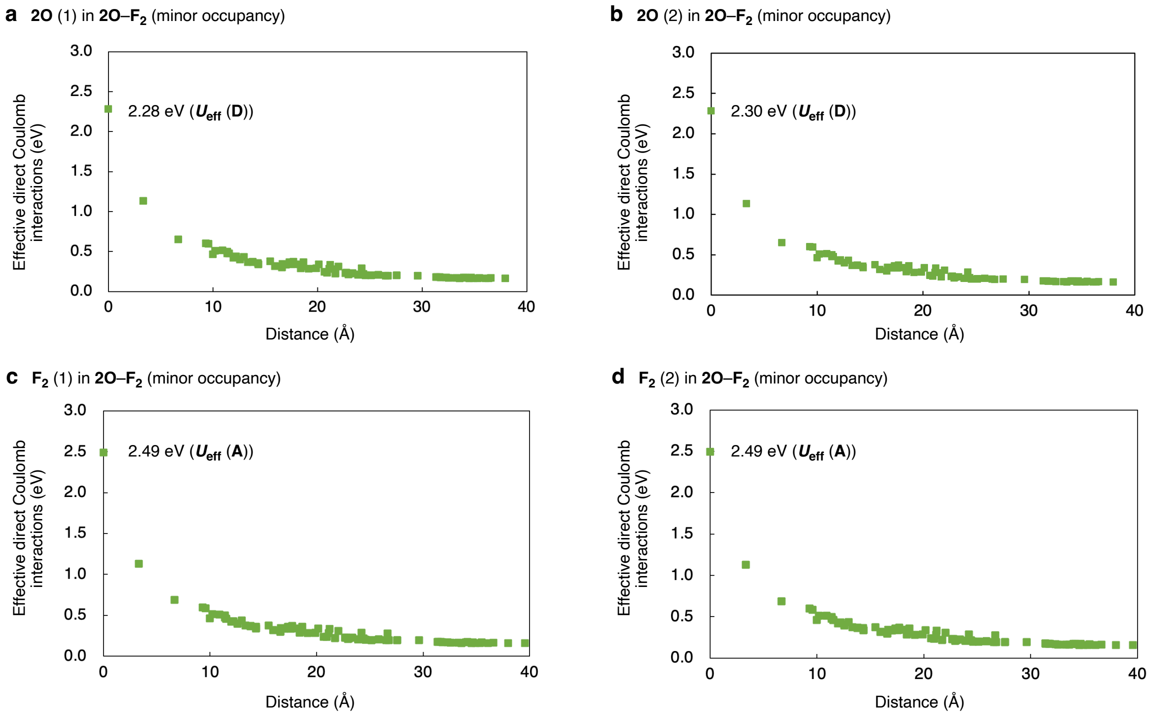

Supplementary Fig. 30: Effective direct Coulomb interactions in a cell of 2O–F_2 in the minor occupancy. Values of donors (**a**,**b**), and acceptors (**c**,**d**).

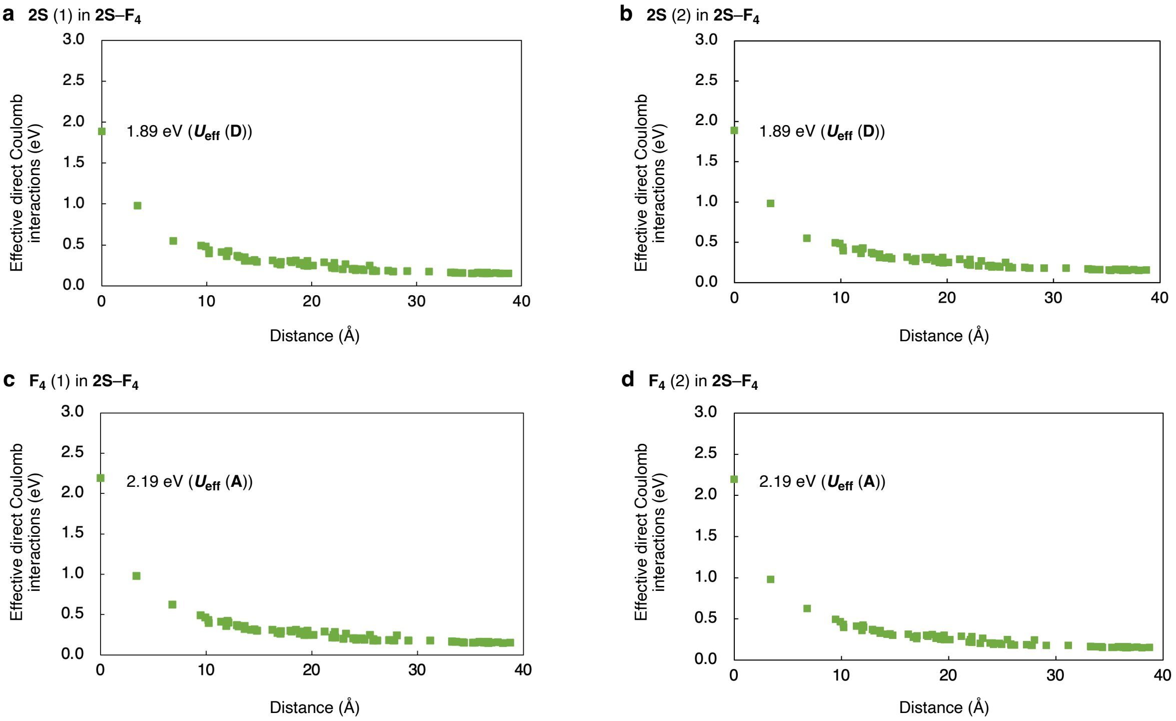

Supplementary Fig. 31: Effective direct Coulomb interactions in a cell of 2S–F_4. Values of donors (**a**,**b**), and acceptors (**c**,**d**).

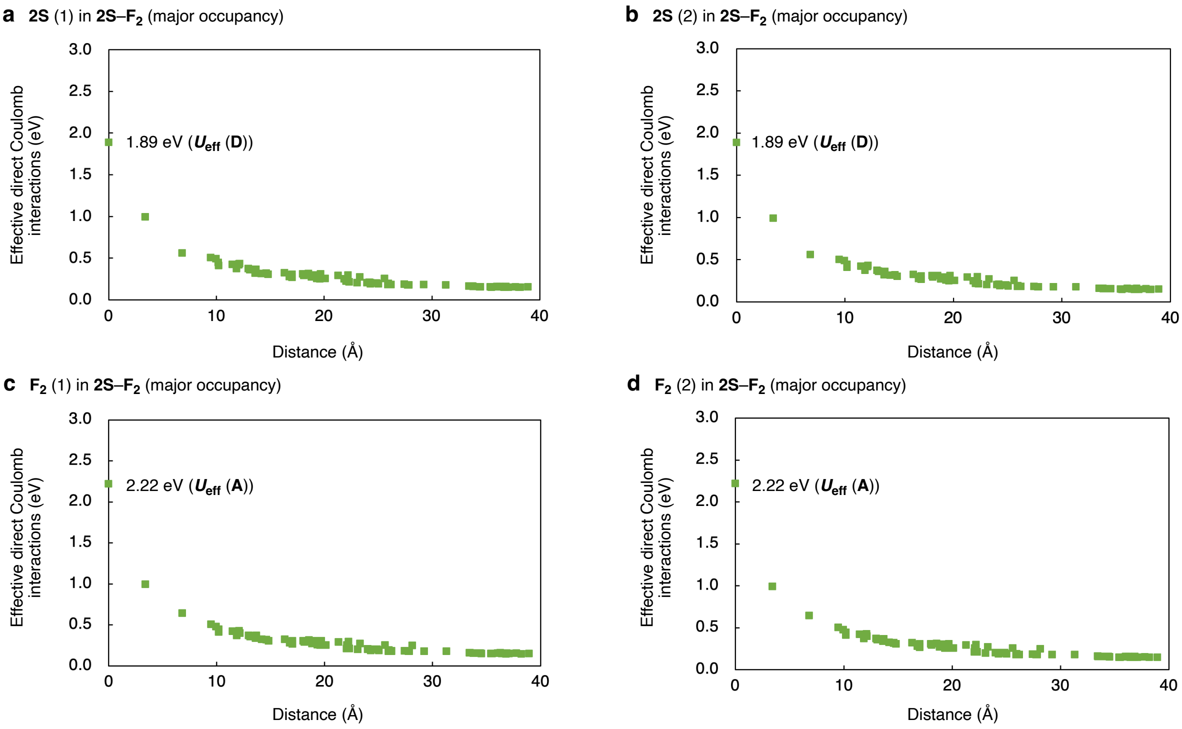

Supplementary Fig. 32: Effective direct Coulomb interactions in a cell of 2S–F_2 in the major occupancy. Values of donors (**a**,**b**), and acceptors (**c**,**d**).

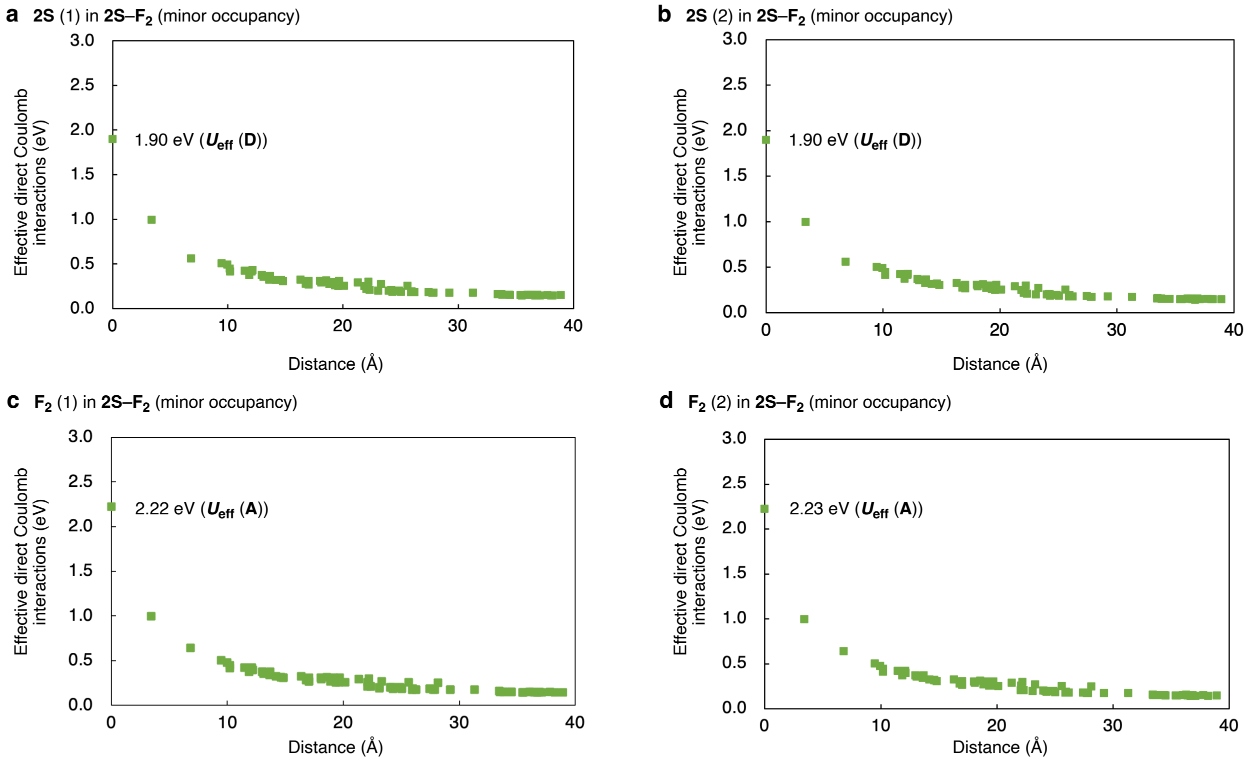

Supplementary Fig. 33: Effective direct Coulomb interactions in a cell of 2S–F$_2$ in the minor occupancy. Values of donors (**a**,**b**), and acceptors (**c**,**d**).

7. Optical properties

Supplementary Note 8: Optical reflection spectroscopy measurements.

The mixed-stack complexes showed multiple sharp peaks in the optical reflectivity spectra along the π-stacking direction in the low-energy region of 0.14–0.19 eV at 293 K (Fig. 6 and Supplementary Fig. 34), except for the nearly neutral **2S–F$_2$**. The optical conductivity spectrum of **2S–F$_4$** along the π-stacking direction, which was derived from the polarized infrared reflectivity spectrum, and Raman spectrum at the excitation wavelength of 532 nm at 293 K are shown in Supplementary Fig. 35b,d. The optical conductivity and Raman spectra likely exhibited the same modes. Considering the inversion center of the single-crystal structure of **2S–F$_4$** categorized to be $P2_1/n$ with uniform π-stacked donors and acceptors along the $C2$ glide symmetry, the Raman-active modes should not be visible in IR, while the IR-active modes should not be visible in Raman. The coincidence of the shapes of the observed IR and Raman spectra indicates the dynamic fluctuating of donor–acceptor-π-stacking dimerization.[24,25] The optical activation of the a_g mode

along the π-stacking direction can be ascribed to the electron-molecular vibration (EMV) couplings.[26,27] To confirm that the EMV coupling effect is responsible for these signals, we performed numerical simulations of the optical activation of a_g modes based on the donor–acceptor dimer model proposed by Painelli, A. and Girlando, A.[28] The calculation requires the frequencies (ω) of the molecular normal modes (Q) and the corresponding g-values defined as $g = \sqrt{2\omega} d\epsilon/dQ$. Initially, we performed DFT calculations for the isolated states of the donor and acceptor molecules to ascertain the initial parameters for the simulation. The eigenfrequencies of molecular vibrations were determined through standard DFT vibrational analysis. The g-value, which are the derivatives of the radical electronic level ϵ_{HOMO} (or ϵ_{LUMO} for neutral acceptor) in terms of Q, are estimated by slightly deforming the molecular structure along Q and calculating the energy shifts of the HOMO or LUMO levels. The quantum chemical calculation package Gaussian16[29] was used for the DFT calculations. The B3LYP function was used for the density functional, with the basis sets 6-311G(d)[30,31,32] for the donor molecule and 6-311G+(d) for the acceptor molecule. Noting that B3LYP tends to overestimate eigenfrequencies ω, the calculated frequencies were corrected by applying the standard scaling factor of 0.96. The values of ω and g vary based on the valence state of the molecule. In the present study, we calculated these values for both the neutral and monovalent ionic states of the donor and acceptor and interpolated for the relevant ionicity states ($+\delta$ for the donor and $-\delta$ for the acceptor; δ: degree of charge transfer; Supplementary Table 4), according to the method outlined in the paper by Painelli, A. and Girlando, A.[28] Using these values as initial parameters, calculations of the dimer model were conducted. The specific parameters used for the calculation are detailed in Supplementary Table 5. By adjusting the calculated values of ω and g for each vibrational mode, as listed in Supplementary Table 4, we achieved good agreement with experimental values, as shown in Supplementary Fig. 35b,c. The adjustment of parameters falls within a reasonable range, affirming that the dimer model accurately reproduced the experimental spectrum.

The optical activation of the a_g mode due to EMV coupling implies that the one-dimensional molecular

stacking is not uniform, contradicting the uniform columnar structure predicted by X-ray structural analysis (Supplementary Table 2 and Supplementary Fig. 10). The activation of the EMV modes hints at dynamic deviations from the average structure within the molecular columns, supporting the dynamic π-dimerization fluctuation. In the unit cell of this crystal, there are two sets of the donor–acceptor pairs, which are linked via inversion symmetry. The transition moments generated by the EMV coupling within a pair couple in either the same direction or in the opposite direction with another pair, resulting in both infrared active and Raman active EMV modes. This explains the coincidence of the shapes of the observed IR and Raman spectra. The slight difference in peak position between the IR and Raman signals can be understood as a Davydov splitting due to the dipole interaction between the pairs.

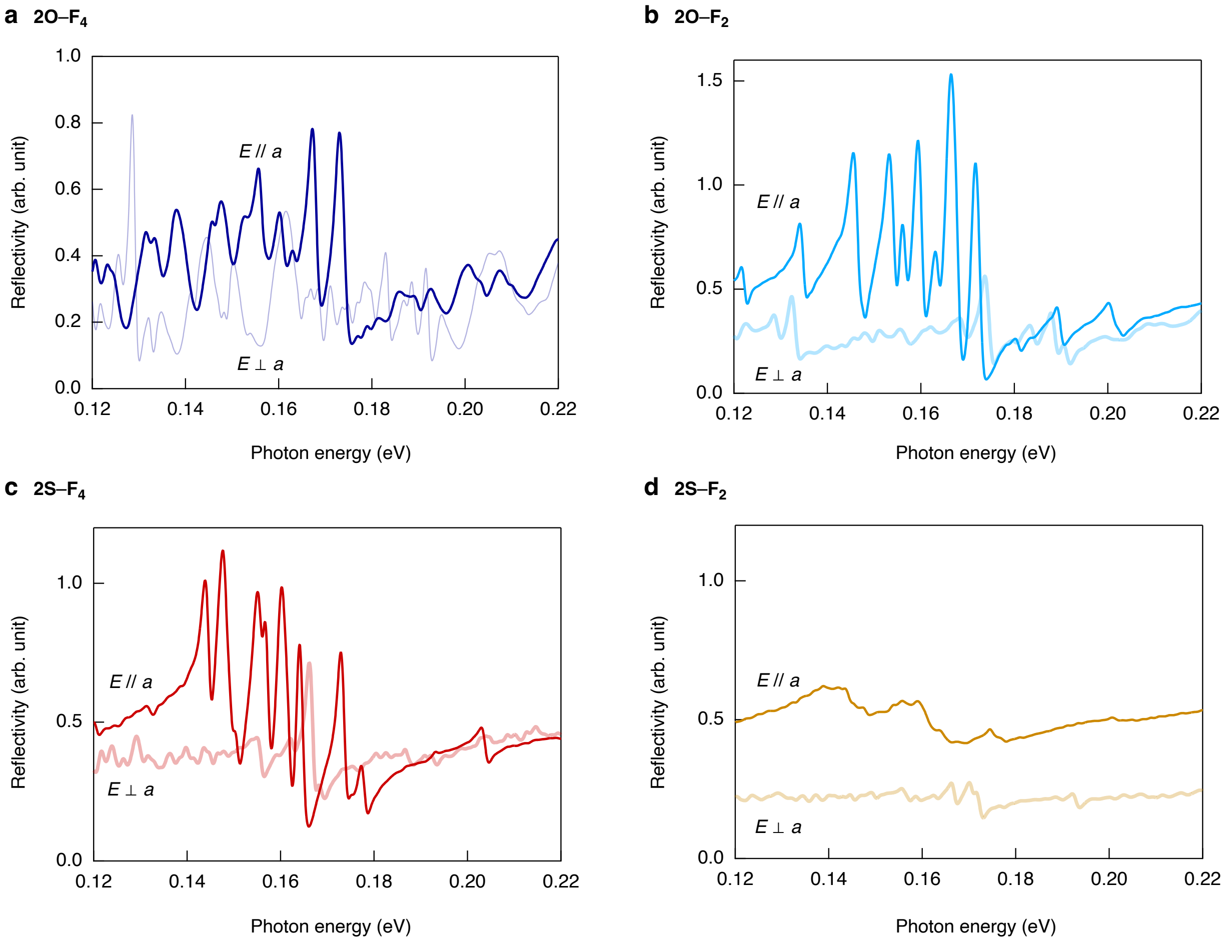

Supplementary Fig. 34: Reflectivity spectra of mixed-stack complexes. Spectra of **2O–F$_4$** (**a**), **2O–F$_2$** (**b**), **2S–F$_4$** (**c**), and **2S–F$_2$** (**d**) at 293 K, measured with the electric field of the light (*E*) parallel (//) and

perpendicular (⊥) to the π-stacking directions. The relatively thin single-crystal **2O–F4** exhibited the stripe-pattern signals for the low-energy region due to the interference effect from the backside.

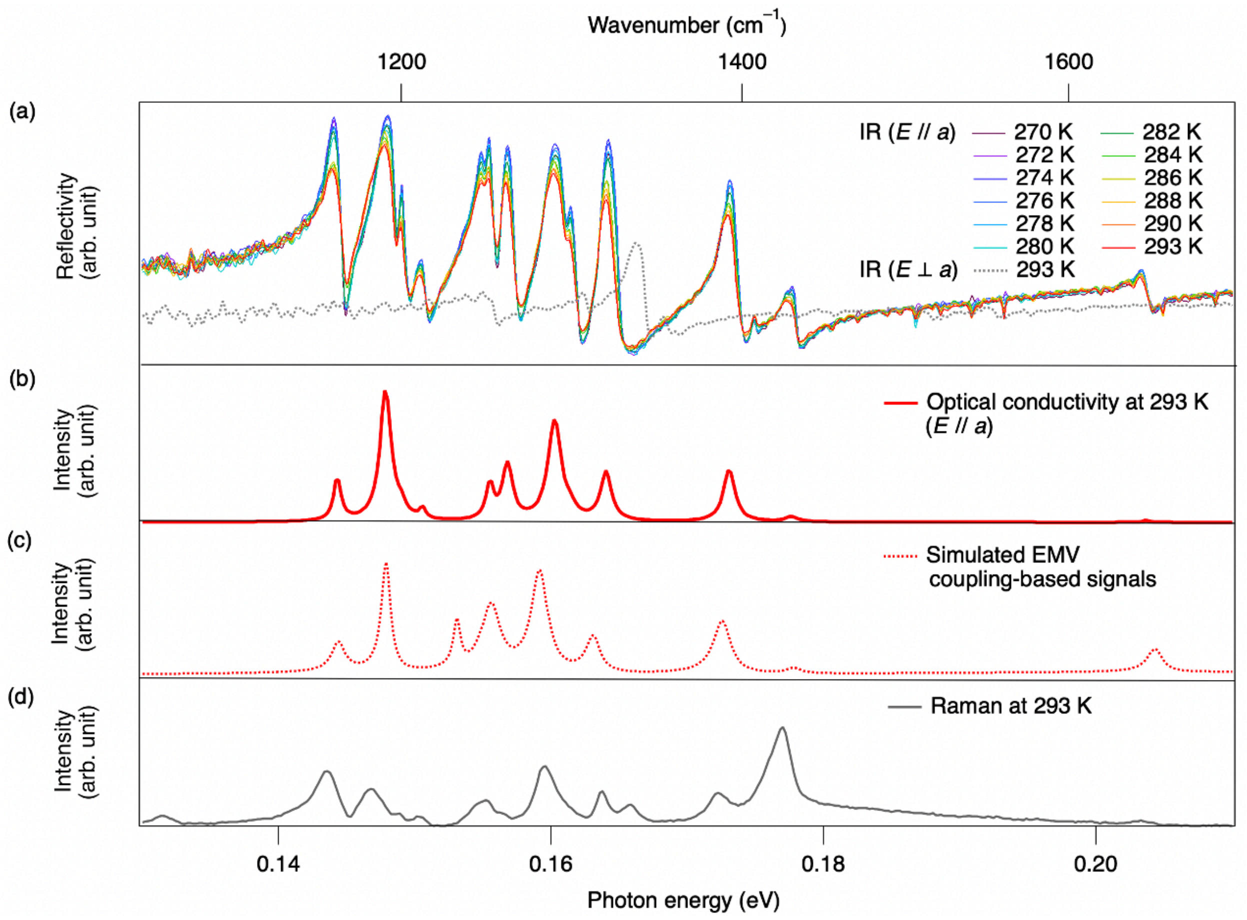

Supplementary Fig. 35: Temperature-dependent polarized reflectivity spectra. a, The spectra were obtained by applying an electric field along the π-stacking direction (solid lines) and perpendicular to the π-stacking direction (a dashed line). **b**, Optical conductivity spectrum derived from the polarized infrared spectrum at 293 K along the π-stacking direction through the Kramers-Kronig transformation. **c**, Simulated EMV coupling-based spectrum. The values of ω and g for both the neutral and monovalent ionic states of the donor and acceptor were calculated and interpolated for the relevant ionicity according to the method outlined in the paper by Painelli, A. and Girlando, A.,[28] as listed in Supplementary Table 4. Using these values as initial parameters, calculations of the dimer model were conducted. The specific parameters in the calculations are listed in Supplementary Table 5. **d**, Raman spectrum at 293 K (Excitation wavelength: 532 nm).

Supplementary Table 4: The fundamental molecular vibration modes of donor and acceptor molecules estimated in the relevant ionicity calculated from their neutral and ionic states.

Entry	Molecule	Wavenumber (cm^{-1})	*g* value (meV)
1	$\mathbf{F_4}$	1608.28	43.85
2	**2S**	1435.71	5.84
3	$\mathbf{F_4}$	1405.09	126.67
4	**2S**	1380.18	12.46
5	**2S**	1335.17	21.54
6	**2S**	1312.94	27.06
7	**2S**	1295.49	18.23
8	$\mathbf{F_4}$	1235.33	47.93
9	**2S**	1156.45	25.70
10	**2S**	1116.31	6.30

Supplementary Table 5: Parameters used in the calculation of the vibronic spectrum of 2S–$\mathbf{F_4}$.

Entry	Molecule	Wavenumber (cm^{-1})	*g* value (meV)	linewidth (cm^{-1})
1	$\mathbf{F_4}$	1650	20	10
2	**2S**	1435	10	10
3	$\mathbf{F_4}$	1395	30	10
4	**2S**	1320	30	10
5	**2S**	1295	50	10
6	**2S**	1260	40	10
7	**2S**	1250	10	10
8	$\mathbf{F_4}$	1245	40	10
9	**2S**	1215	60	10
10	**2S**	1170	30	10

8. Electrical conductivities

Supplementary Note 9: Electrical resistivity measurements.

The current–voltage (*I*–*V*) characteristics by a two-probe method at room temperature from −10 to 10 V for **2O–F**$_4$/**2S–F**$_2$, −3 to 3 V for **2S–F**$_2$, and −1.2 to 1.5 V for **2S–F**$_4$ (Supplementary Fig. 36) confirmed the ohmic behaviors for the *V* ranges. Within the ohmic regions, the temperature (*T*)-dependent resistance (*R*) of the sample was measured by a four-probe method upon cooling the electrode from 340 to 200 K and subsequent heating to 340 K by 1.0–1.5 K min^{-1} (Figs. 7 and 8a).

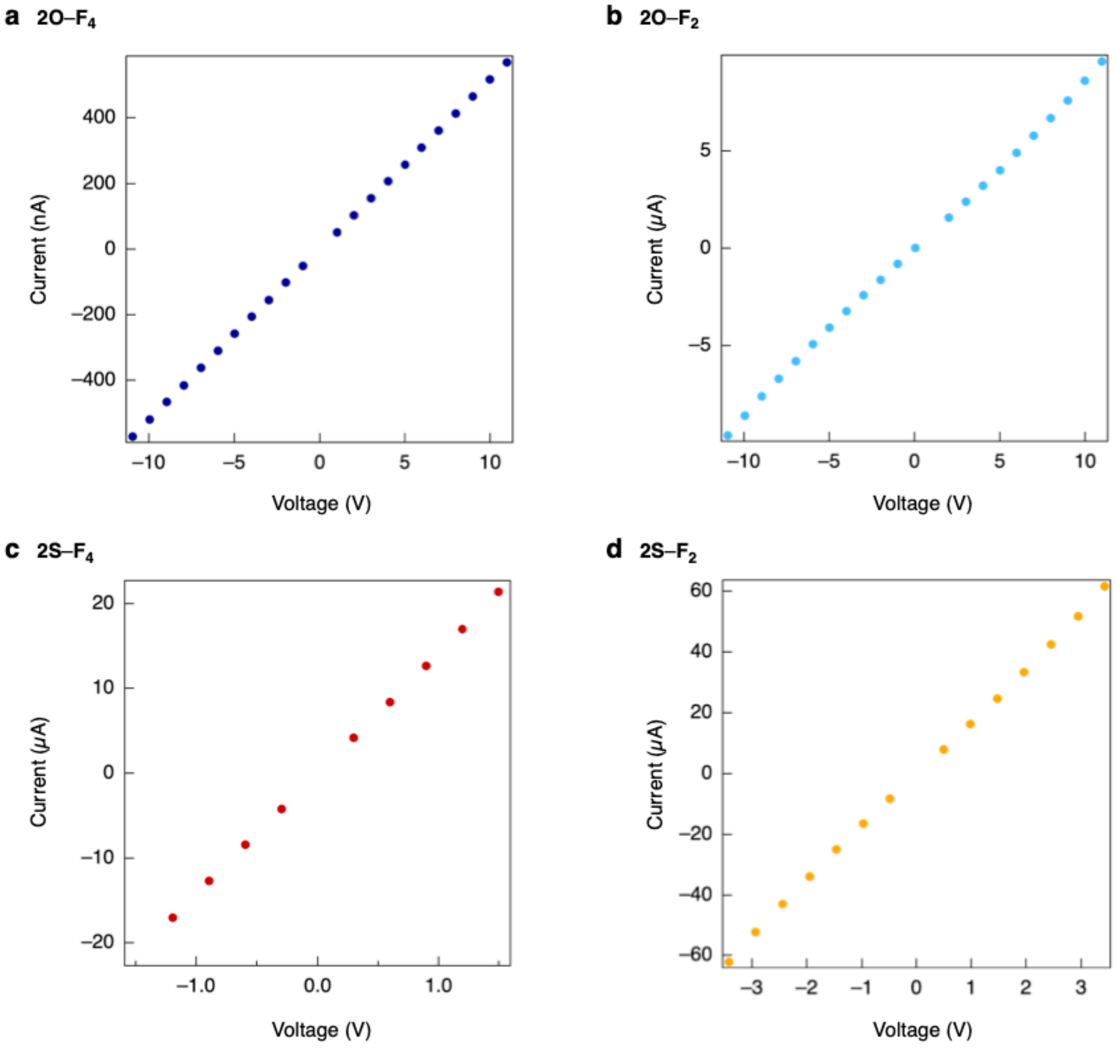

Supplementary Fig. 36: *I*–*V* curve of mixed-stack complexes. a, 2O–F$_4$. **b**, 2O–F$_2$. **c**, 2S–F$_4$. **d**, 2S–F$_2$. The measurements were performed at 293 K.

9. Magnetic properties

Supplementary Note 10: ESR measurements.

The X-band (~9.4 GHz) continuous wave electron spin resonance (ESR) experiments were performed on single-crystal **2O–F**$_4$, **2O–F**$_2$, **2S–F**$_4$, and **2S–F**$_2$ (Fig. 8d,e, and Supplementary Figs. 37–39). From the ESR spectra, the spin susceptibility values χ_{spin} were calculated by the following equation:

$$\chi_{\mathrm{spin}} \propto I_{\mathrm{m}} \times (\Delta\mathbf{B}_{\mathrm{pp}})^2 \qquad (1)$$

, where maximum intensity I_{m}, the peak-to-peak width $\Delta\mathbf{B}_{\mathrm{pp}}$, and resonance center $\mathbf{B}_0$ were determined by fitting to the equation of Lorentzian as follows.

$$I(B) = a \times \frac{16 \times I_m \times (\mathbf{B}_0 - \mathbf{B})/(\Delta\mathbf{B}_{\mathrm{pp}} / 2)}{[3 + \{\frac{\mathbf{B}_0 - \mathbf{B}}{\Delta\mathbf{B}_{\mathrm{pp}}} / 2\}^2]^2} \qquad (2).$$

Spectra of **2S–F**$_4$ were finely fitted to sum of two Lorentzian curves, and $I_{\mathrm{m\_a}}$, $I_{\mathrm{m\_b}}$, $\Delta\mathbf{B}_{\mathrm{pp\_a}}$, $\Delta\mathbf{B}_{\mathrm{pp\_b}}$, $\mathbf{B}_{0\_\mathrm{a}}$, and $\mathbf{B}_{0\_\mathrm{b}}$ were determined by using the equation of Lorentzian in the differential form as follows.

$$I(B) = a \times \frac{16 \times I_{m\_\mathrm{a}} \times (\mathbf{B}_{0\_\mathrm{a}} - \mathbf{B})/(\Delta\mathbf{B}_{\mathrm{pp\_a}} / 2)}{[3 + \{\frac{\mathbf{B}_{0\_\mathrm{a}} - \mathbf{B}}{\Delta\mathbf{B}_{\mathrm{pp\_a}}} / 2\}^2]^2} + b \times \frac{16 \times I_{m\_\mathrm{b}} \times (\mathbf{B}_{0\_\mathrm{b}} - \mathbf{B})/(\Delta\mathbf{B}_{\mathrm{pp\_b}} / 2)}{[3 + \{\frac{\mathbf{B}_{0\_\mathrm{b}} - \mathbf{B}}{\Delta\mathbf{B}_{\mathrm{pp\_b}}} / 2\}^2]^2} \qquad (3).$$

The determined parameters as a function of temperatures are shown in Fig. 8d,e and Supplementary Fig. 39.

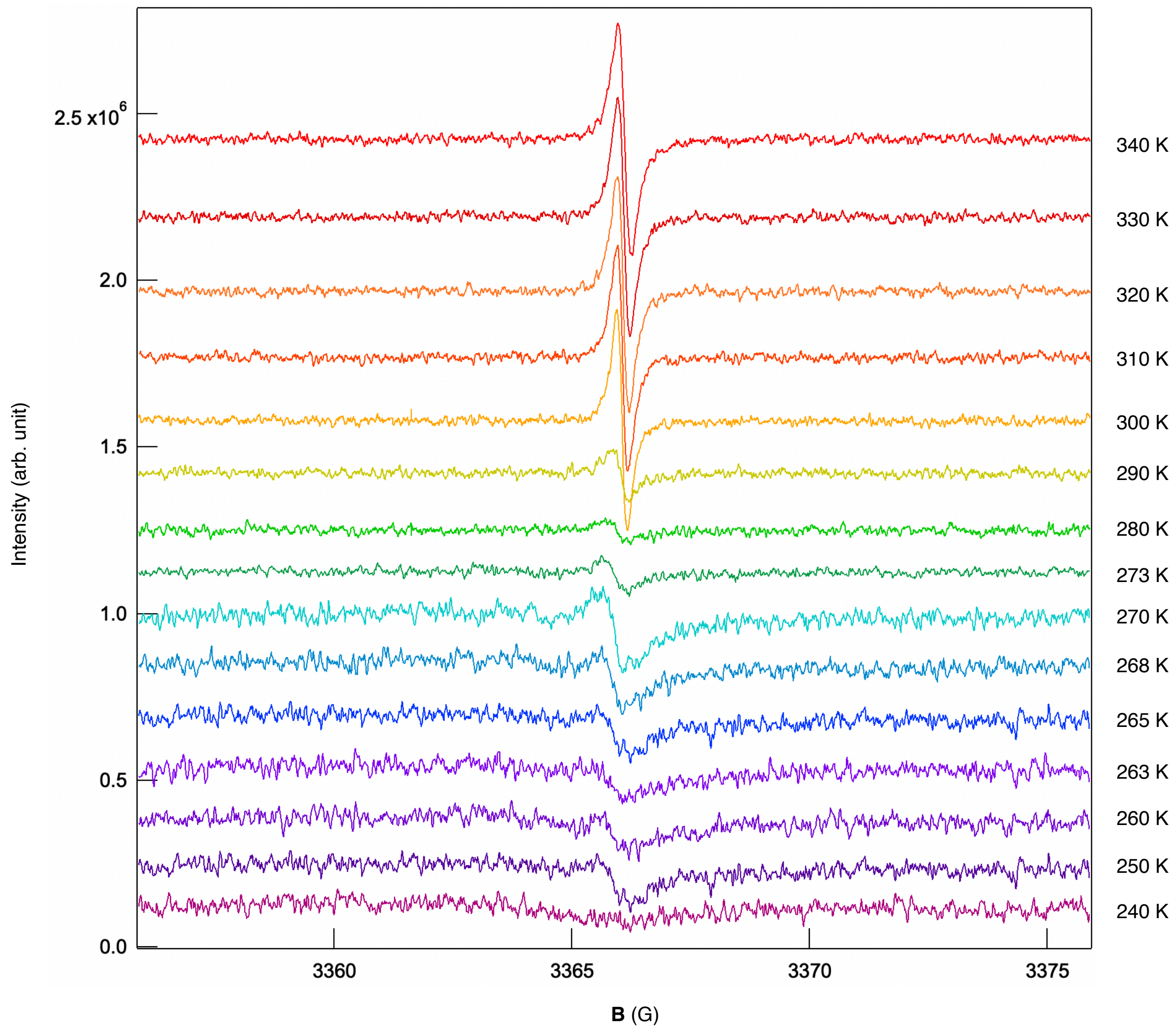

Supplementary Fig. 37: Temperature-dependent ESR spectra of 2S–F_4.

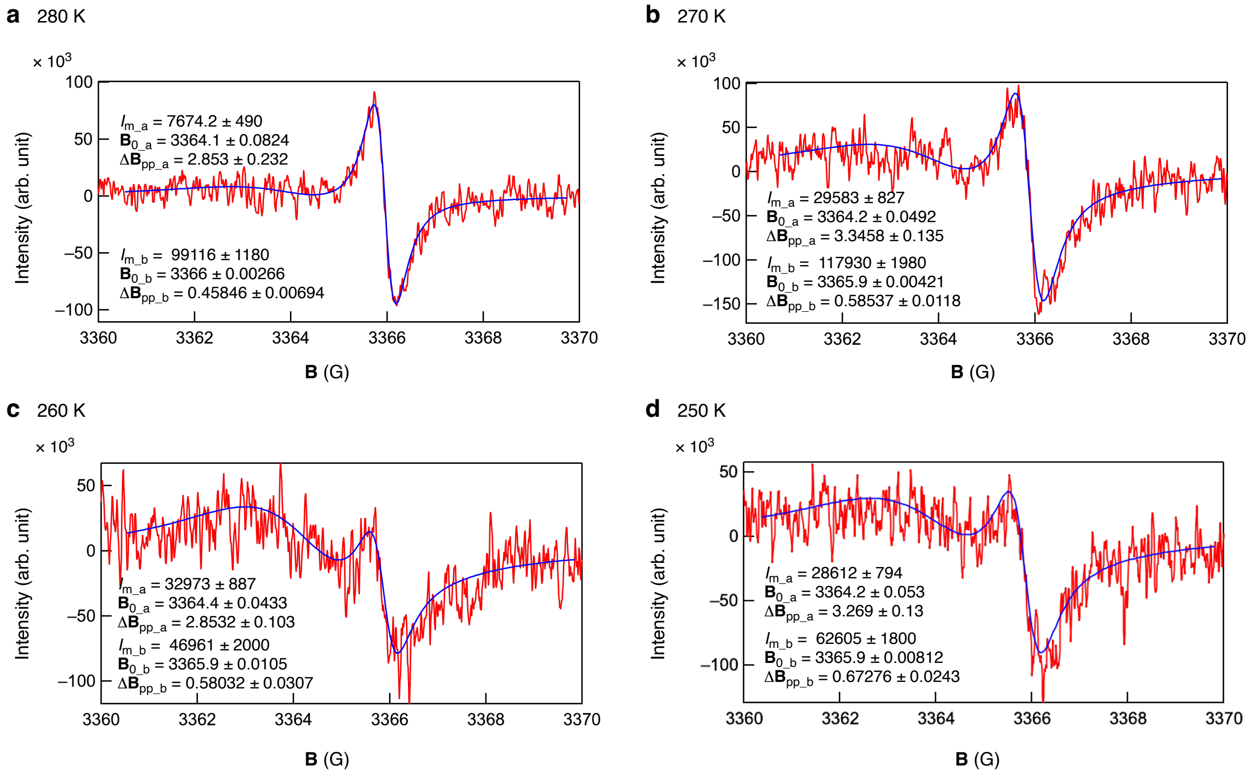

Supplementary Fig. 38: Enlarged ESR spectra of 2S–F$_4$. a, 280 K. **b**, 270 K. **c**, 260 K. **d**, 250 K. Blue lines show fitting curves composed of two Lorentzian curves. The spectrum at 280 K was obtained by accumulating signals for one-fourth of scans of those below 280 K.

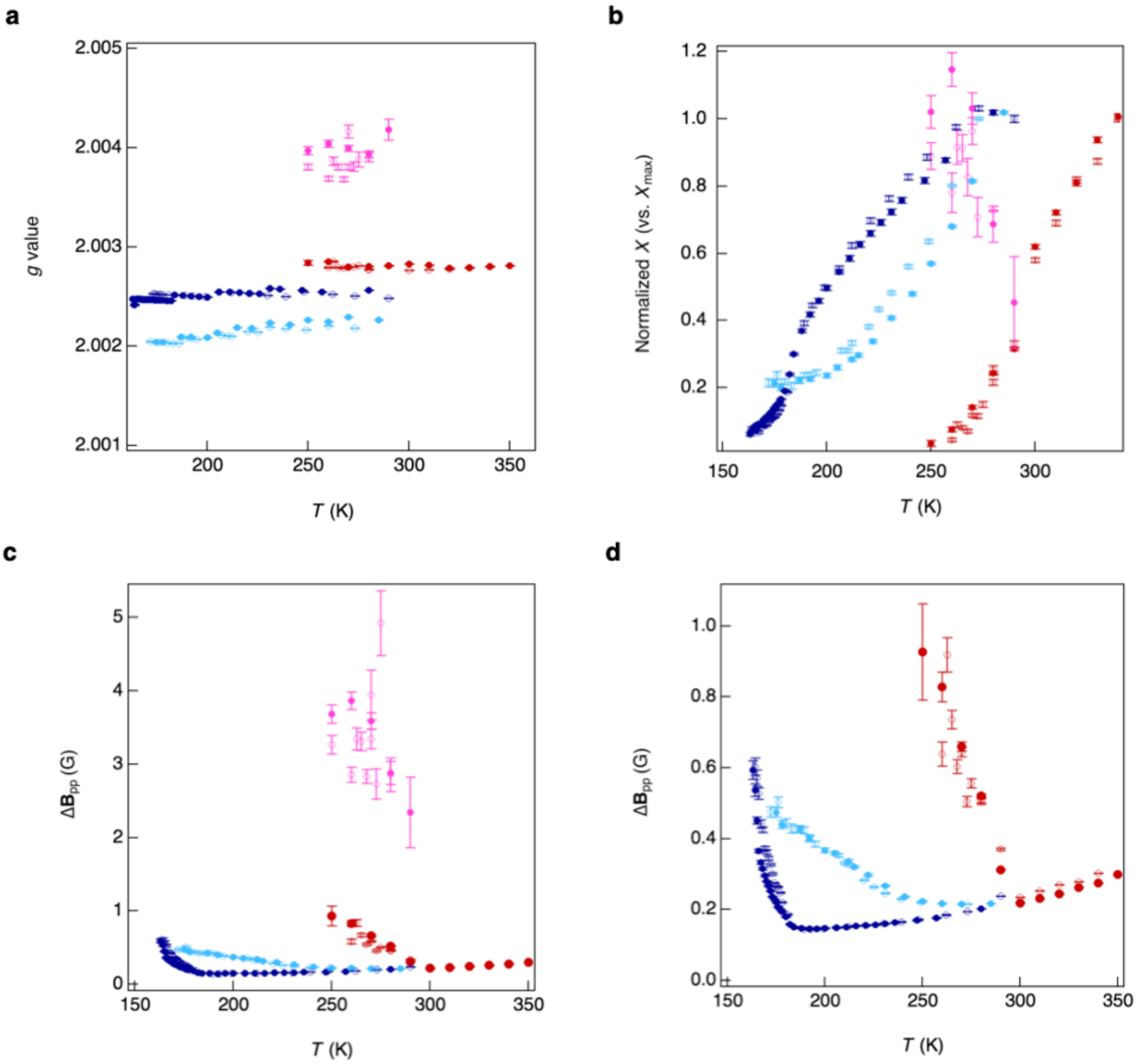

Supplementary Fig. 39: Parameters for ESR spectra of mixed-stack complexes. Parameters of **2O–F$_4$** (blue), **2O–F$_2$** (aqua), and major signals for **2S–F$_4$** (red), and minor signals for **2S–F$_4$** (pink) upon the cooling (blank circles) and heating (filled circles) processes. Temperature dependence of *g* value (**a**), normalized χ_{spin} (**b**) relative to the maximum value (for the major signals in **2S–F$_4$**), ΔB_{pp} (**c**,**d**) are shown (**d**: the enlarged chart of **c**).

Supplementary Note 11: Static magnetic susceptibility measurements

The static magnetic susceptibility of polycrystals **2O–F$_4$** and **2S–F$_4$** was measured applying the static magnetic field of 10,000 O$_e$ (Supplementary Fig. 40). The χ_{exp} values were adjusted by subtracting the value at 100 and 80 K for **2O–F$_4$** and **2S–F$_4$**, respectively, to be 2.6×10^{-4} and 1.2×10^{-4} emu mol^{-1} at 293 K, respectively. The χ values were relatively high compared to those of previously reported mixed-stack complexes,[33,34,35] most of which are in nearly nonmagnetic states. These values are comparable to those of typical organic conductors,[22] emphasizing the presence of abundant conductive electrons.

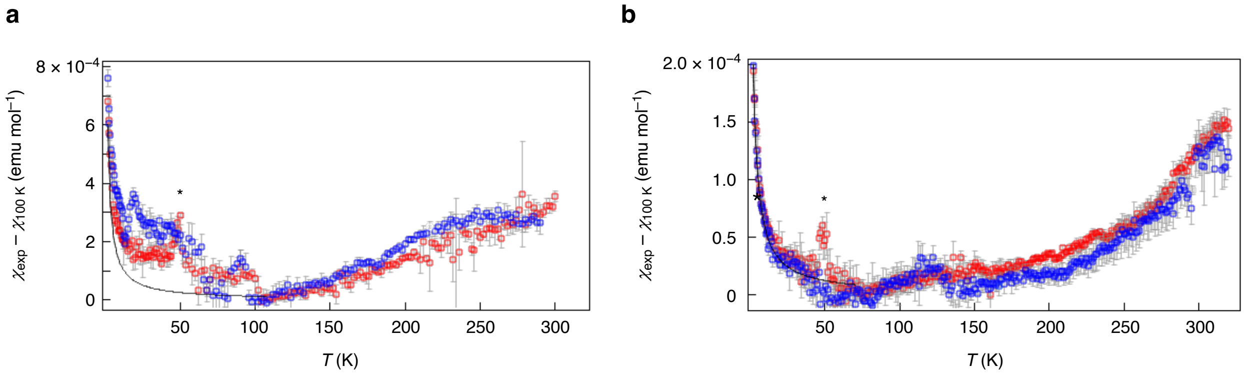

Supplementary Fig. 40: Temperature-dependent susceptibility of mixed-stack complexes. a, **2O–F$_4$**. **b**, **2S–F$_4$**. The susceptibility upon the cooling (blue) and heating (red) processes were shown. Black lines are fitting curves of χ_{CW}. Asterisks indicate the anomaly attributed to the presence of molecular oxygen in the sample holder.

10. NMR spectra

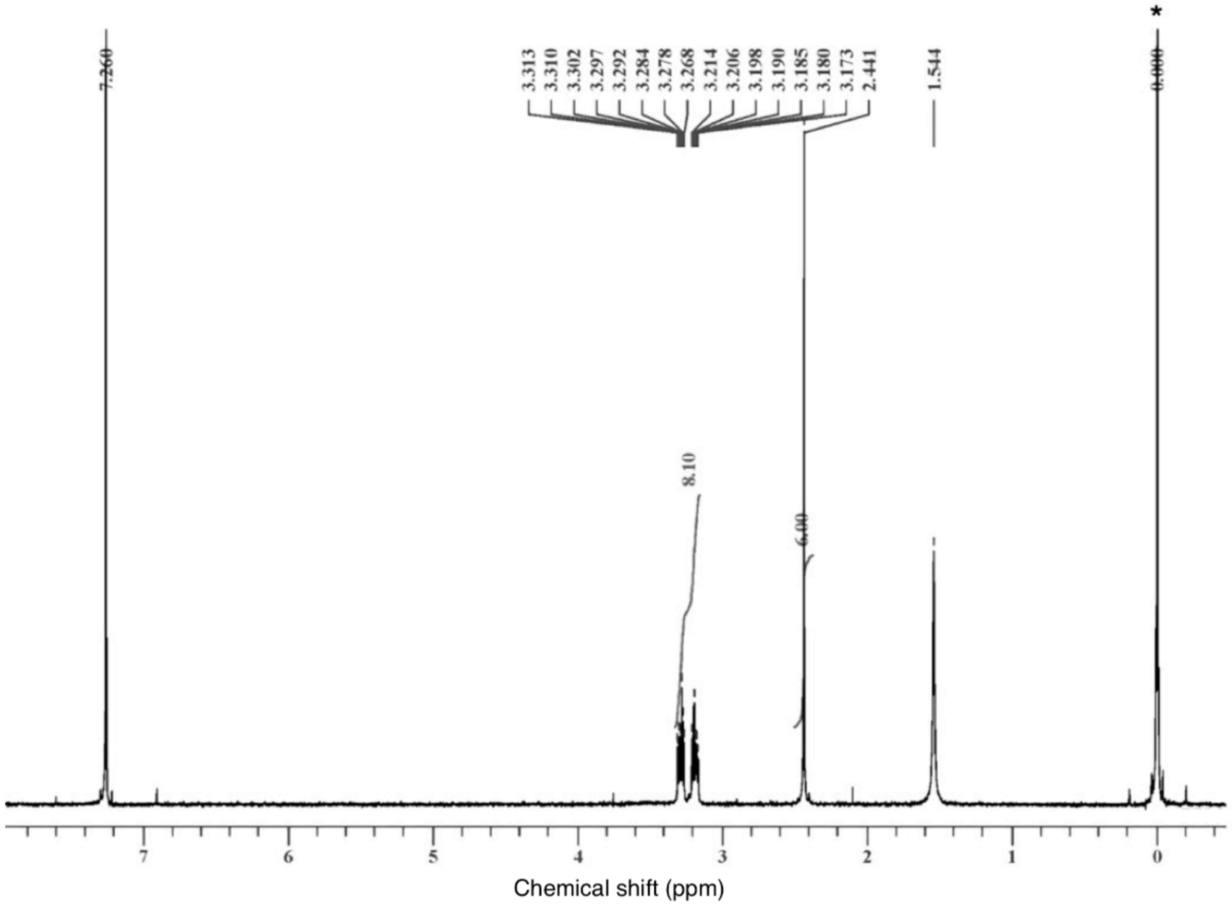

Supplementary Fig. 41: ^{1}H NMR spectrum of 2S in $CDCl_3$. The signal for $Si(CH_3)_4$ used for the internal standard was shown with an asterisk.

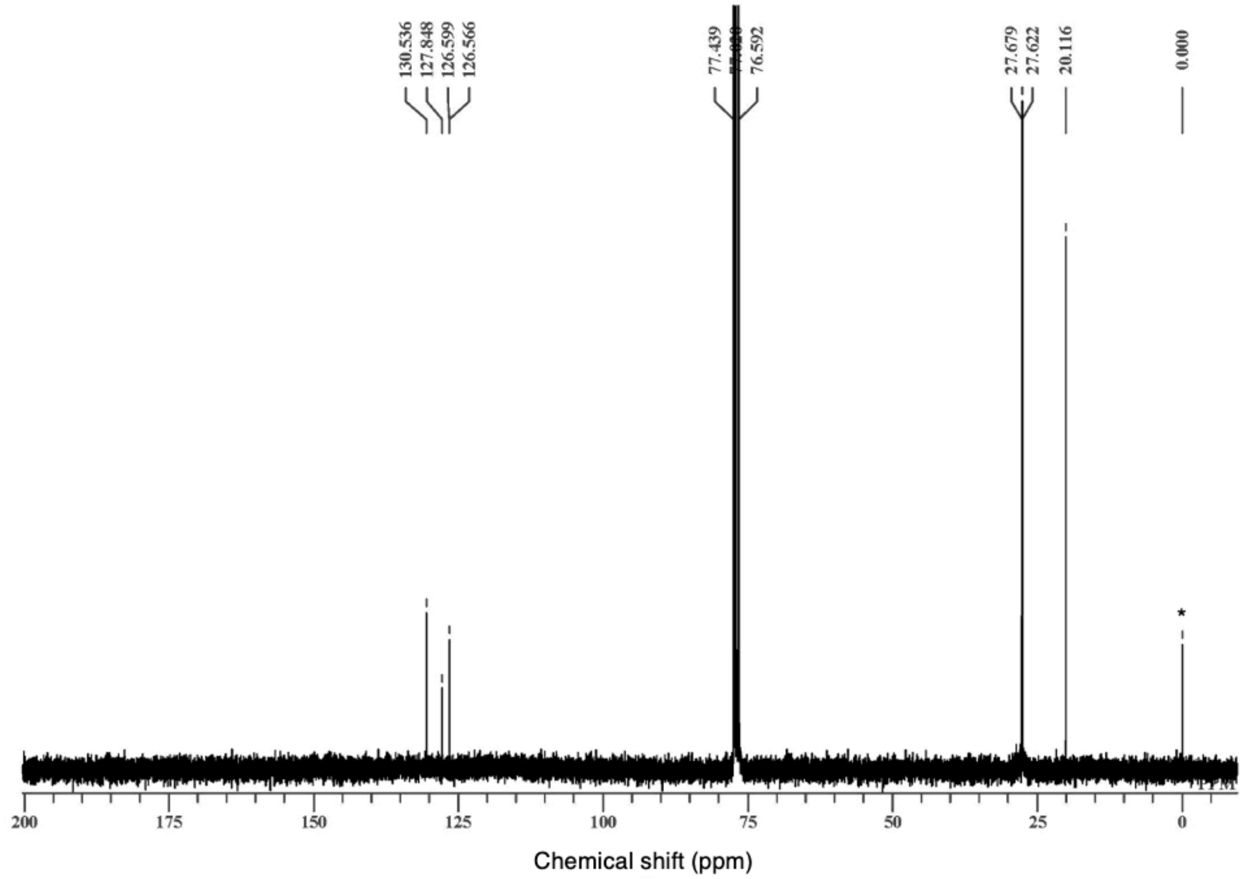

Supplementary Fig. 42: ^{13}C NMR spectrum of 2S in $CDCl_3$. The signal for $Si(CH_3)_4$ used for the internal standard was shown with an asterisk.

11. Coordinates of optimized structures

Supplementary Table 6: The geometry of optimized structure for neutral 2S. The calculation was performed by Gaussian (B3LYP, 6-31G+(d)).

C1	4.0228	3.9680	0.3304
C2	3.3195	4.8232	1.1597
C3	4.0070	5.0995	2.3940
C4	1.5071	6.8425	1.7958
H5	2.1672	7.6565	1.4809
H6	0.4703	7.1592	1.6351
C7	1.7233	6.4966	3.2641
H8	1.1056	5.6417	3.5543
H9	1.4497	7.3489	3.8966
S10	5.5429	3.5003	1.0494
C11	3.6193	3.4720	-0.9884
C12	2.9108	2.3199	-1.2793
C13	2.7076	2.1287	-2.6918
C14	1.1432	0.2125	-0.8052
H15	0.2373	0.8072	-0.9570
H16	0.9173	-0.5793	-0.0820
C17	1.6243	-0.4007	-2.1146
H18	2.5635	-0.9415	-1.9661
H19	0.8782	-1.1068	-2.4969
S20	4.0452	4.3363	-2.4426
C21	3.2539	3.1512	-3.4477
C22	5.2348	4.4652	2.4688
S23	3.2481	3.3133	-5.2047

S24	6.3920	4.5155	3.7998
C25	7.4047	5.9878	3.3547
H26	7.9108	5.8355	2.3982
H27	6.7876	6.8887	3.3182
H28	8.1524	6.0937	4.1468
C29	1.6442	4.1710	-5.4923
H30	1.6335	5.1473	-5.0016
H31	1.5625	4.3085	-6.5749
H32	0.8079	3.5614	-5.1418
S33	1.8056	0.8140	-3.4771
S34	2.4308	1.2145	0.0278
S35	1.7043	5.3964	0.6890
S36	3.4778	6.1829	3.7001

Supplementary Table 7: The geometry of neutral 2S with constrained torsional angle $|\theta_1|$ (defined in Supplementary Fig. 4a) to be 180°. The calculation was performed by Gaussian (B3LYP, 6-31G+(d)).

S1	1.0635	0.8431	6.9634
S2	1.9869	4.6387	5.2369
S3	2.8445	1.8820	3.2238
S4	0.5690	3.6422	8.0934
C5	1.7841	2.9201	5.6445
C6	1.7652	0.6213	5.4054
C7	2.0674	1.8077	4.8181
C8	1.1864	2.6257	6.8442
C9	2.9367	4.6345	3.6534

H10	2.7960	5.6449	3.2824
H11	3.9884	4.4880	3.8533
C12	2.4110	3.5886	2.6797
H13	2.8926	3.6420	1.7081
H14	1.3423	3.6933	2.5373
C15	2.1132	4.0142	9.0685
H16	2.4853	3.0515	9.4029
H17	1.7587	4.5874	9.9079
H18	2.8517	4.5385	8.4757
S19	2.4713	-0.4506	3.0881
S20	1.9784	-4.6286	3.8407
S21	1.2525	-2.6859	6.6262
S22	2.9219	-2.8632	1.2797
C23	2.0043	-2.8506	3.9299
C24	1.9355	-0.6655	4.7241
C25	1.7679	-2.0032	5.0690
C26	2.4075	-2.2201	2.7989
C27	1.2691	-5.1968	5.4400
H28	1.5268	-6.2509	5.4710
H29	0.1921	-5.1101	5.4225
C30	1.8708	-4.4259	6.5958
H31	1.5415	-4.8013	7.5599
H32	2.9539	-4.4554	6.5680
C33	1.2843	-3.0593	0.4097
H34	0.8564	-2.0634	0.3663

H35	1.5556	-3.3884	-0.5781
H36	0.6253	-3.7486	0.9213

Supplementary Table 8: The geometry of optimized structure for radical cation 2S$^{•+}$. The calculation was performed by Gaussian (UB3LYP, 6-31G+(d)).

C1	3.3129	4.2093	0.4156
C2	3.1834	5.5683	0.8268
C3	3.7039	5.8146	2.1208
C4	2.7418	8.3764	0.6307
H5	3.7485	8.7410	0.4136
H6	2.0189	9.0442	0.1498
C7	2.4913	8.3113	2.1296
H8	1.5000	7.9127	2.3615
H9	2.5696	9.3141	2.5620
S10	4.1595	3.2771	1.6730
C11	2.8214	3.5610	-0.7497
C12	3.2276	2.3380	-1.3564
C13	2.4205	1.9552	-2.4543
C14	4.5446	-0.1455	-1.6229
H15	3.8059	-0.7506	-1.0917
H16	5.5347	-0.5856	-1.4623
C17	4.2379	-0.0679	-3.1119
H18	4.9560	0.5661	-3.6383
H19	4.2750	-1.0700	-3.5521
S20	1.4157	4.2173	-1.6097

C21	1.3942	2.8774	-2.7146
C22	4.2467	4.6634	2.7130
S23	0.2532	2.7096	-4.0105
S24	4.9255	4.6364	4.3089
C25	5.4977	2.9103	4.4914
H26	4.6603	2.2095	4.4456
H27	6.2561	2.6649	3.7436
H28	5.9483	2.8638	5.4857
C29	-0.8077	4.1859	-3.8259
H30	-0.2290	5.1052	-3.9480
H31	-1.5384	4.1146	-4.6351
H32	-1.3335	4.1774	-2.8679
S33	2.5296	0.4969	-3.4491
S34	4.6876	1.5024	-0.8267
S35	2.4723	6.7801	-0.2410
S36	3.7814	7.3472	2.9960

Supplementary Table 9: The geometry of optimized structure for neutral F_4. The calculation was performed by Gaussian (B3LYP, 6-31G+(d)).

C1	-0.9377	-0.3076	1.0919
C2	0.5063	-0.4011	1.093
C3	1.1799	0.8796	1.0942
C4	0.5152	2.0649	1.0943
C5	-0.9288	2.1584	1.0932
C6	-1.6024	0.8777	1.092

C7	-0.9597	4.6449	1.0945
N8	-0.5225	5.724	1.0954
C9	-3.0336	3.4831	1.0922
N10	-4.1821	3.6745	1.0914
C11	0.5371	-2.8876	1.0917
N12	0.0999	-3.9667	1.0908
C13	2.6111	-1.7258	1.094
N14	3.7595	-1.9172	1.0948
F15	1.2301	3.191	1.0955
F16	2.5136	0.9022	1.0953
F17	-2.9361	0.8551	1.0909
F18	-1.6526	-1.4337	1.0907
C19	-1.6078	3.3699	1.0933
C20	1.1853	-1.6126	1.0929

Supplementary Table 10: The geometry of optimized structure for radical anion $F_4^{\bullet-}$. The calculation was performed by Gaussian (UB3LYP, 6-31G+(d)).

C1	-0.9377	-0.3076	1.0919
C2	0.5063	-0.4011	1.0930
C3	1.1799	0.8796	1.0942
C4	0.5152	2.0649	1.0943
C5	-0.9288	2.1584	1.0932
C6	-1.6024	0.8777	1.0920
C7	-0.9597	4.6449	1.0945
N8	-0.5225	5.7240	1.0954

C9	-3.0336	3.4831	1.0922
N10	-4.1821	3.6745	1.0914
C11	0.5371	-2.8876	1.0917
N12	0.0999	-3.9667	1.0908
C13	2.6111	-1.7258	1.0940
N14	3.7595	-1.9172	1.0948
F15	1.2301	3.1910	1.0955
F16	2.5136	0.9022	1.0953
F17	-2.9361	0.8551	1.0909
F18	-1.6526	-1.4337	1.0907
C19	-1.6078	3.3699	1.0933
C20	1.1853	-1.6126	1.0929

Supplementary Table 11: The geometry of optimized structure for neutral F_2. The calculation was performed by Gaussian (B3LYP, 6-31G+(d)).

C1	-0.9176	-0.3443	1.0919
C2	0.5256	-0.3691	1.0931
C3	1.1716	0.9297	1.0943
C4	0.495	2.1015	1.0944
C5	-0.9482	2.1263	1.0931
C6	-1.5942	0.8275	1.0919
C7	-0.9299	4.5682	1.0944
N8	-0.3408	5.5727	1.0954
C9	-3.0646	3.4636	1.092
N10	-4.2121	3.6617	1.0911

C11	0.5073	-2.811	1.0918
N12	-0.0818	-3.8155	1.0908
C13	2.642	-1.7064	1.0943
N14	3.7895	-1.9045	1.0951
F15	2.5116	0.9545	1.0955
F16	-2.9342	0.8027	1.0907
C17	-1.6434	3.3289	1.0931
C18	1.2208	-1.5716	1.0931
H19	1.0527	3.0318	1.0953
H20	-1.4753	-1.2746	1.0909

Supplementary Table 12: The geometry of optimized structure for radical anion $F_2^{\bullet-}$. The calculation was performed by Gaussian (UB3LYP, 6-31G+(d)).

C1	-0.8893	-0.3368	1.0934
C2	0.5364	-0.3736	1.0935
C3	1.1539	0.9091	1.0931
C4	0.4667	2.0941	1.0928
C5	-0.9590	2.1308	1.0928
C6	-1.5765	0.8481	1.0931
C7	-0.9509	4.5932	1.0921
N8	-0.3445	5.5940	1.0918
C9	-3.0822	3.5076	1.0924
N10	-4.2355	3.7027	1.0924
C11	0.5283	-2.8360	1.0941
N12	-0.0781	-3.8368	1.0944

C13	2.6596	-1.7504	1.0938
N14	3.8129	-1.9454	1.0939
F15	2.5116	0.9692	1.0931
F16	-2.9342	0.7880	1.0931
C17	-1.6705	3.3705	1.0924
C18	1.2479	-1.6133	1.0938
H19	1.0321	3.0201	1.0925
H20	-1.4547	-1.2629	1.0937

12. Supplementary references

1. Kameyama, R., Fujino, T., Dekura, S., Kawamura, M., Ozaki, T. & Mori, H. The simplest model for doped poly(3,4-ethylenedioxythiophene) (PEDOT): single-crystalline EDOT dimer radical cation salts. *Chem. Eur. J.* **27**, 6696–6700 (2021).
2. Cortizo-Lacalle, D., Howells, C. T., Gambino, S., Vilela, F., Vobecka, Z., Findlay, N. J., Inigo, A. R., Thomson, S. A. J., Skabara, P. J. & Samuel, I. D. W. BODIPY-based conjugated polymers for broadband light sensing and harvesting applications. *J. Mater. Chem. A* **22**, 14119–14126 (2012).
3. Seino, Y., Nakamura, H., Mori, H., Fujino, T., Dekura, S. & Kameyama, R. Conductive oligomer, conductive composition, conductive aid, and condenser electrode, transparent electrode, battery electrode, or capacitor electrode formed using said conductive composition. *WO2020262443*, Publication Date 30/12/2020.
4. Uekusa, T., Sato, R., Yoo, D., Kawamoto, T. & Mori, T. Transistor characteristics of charge-transfer complexes observed across a neutral–ionic transition. *ACS Appl. Mater. Interfaces* **12**, 24174−24183 (2020).
5. Sato, R., Kawamoto, T. & Mori, T. Asymmetrical hole/electron transport in donor–acceptor mixed-stack

cocrystals. *J. Mater. Chem. C* **7**, 567–577 (2019).

6. Gaussian 09, Revision D.01, Gaussian, Inc., Wallingford CT, 2016. https://gaussian.com/.

7. Becke, A. D. Density-functional exchange-energy approximation with correct asymptotic behavior. *Phys. Rev. A* **38**, 3098–3100 (1988).

8. Becke, A. D. Density-functional thermochemistry. III. The role of exact exchange. *J. Chem. Phys.* **98**, 5648–5652 (1993).

9. Lee, C., Yang, W. & Parr R. G. Development of the Colle-Salvetti correlation-energy formula into a functional of the electron density. *Phys. Rev. B* **37**, 785–789 (1988).

10. Ditchfield, R., Hehre, W. J. & Pople, J. A. Self-consistent molecular-orbital methods. IX. An extended Gaussian-type basis for molecular-orbital studies of organic molecules. *J. Chem. Phys*. **54**, 724–728 (1971).

11. Hehre, W. J., Ditchfield, R. & Pople, J. A. Self—consistent molecular orbital methods. XII. further extensions of Gaussian—type basis sets for use in molecular orbital studies of organic molecules. *J. Chem. Phys*. **56**, 2257–2261 (1972).

12. Hariharan, P. C. & Pople, J. A. Accuracy of AH_n equilibrium geometries by single determinant molecular orbital theory. *Mol. Phys.* **27**, 209–214 (1974).

13. GaussView 5.0, Version 5.0.8, 2008, Semichem Inc., Shawnee Mission, KS. https://gaussian.com/.

14. Onozuka, K., Fujino, T., Kameyama, R., Dekura, S., Yoshimi, K., Nakamura, T., Miyamoto, T., Yamakawa, T., Okamoto, H., Sato, H., Ozaki, T. & Mori, H. Metallic state of a mixed-sequence oligomer salt that models doped PEDOT family. *J. Am. Chem. Soc.* **145**, 15152–15161 (2023).

15. Kato, Y., Matsumoto, H. & Mori, T. Absence of HOMO/LUMO Transition in charge-transfer complexes of thienoacenes. *J. Phys. Chem. A* **125**, 146–153 (2021).

16. Sheldrick, G. M. *SHELXT*- Integrated space-group and crystal-structure determination. *Acta Cryst.* **A71**, 3–8 (2015).

17. Dolomanov, O. V., Bourhis, L. J., Gildea, R. J., Howard, J. A. K. & Puschmann, H. *OLEX2*: A complete structure solution, refinement and analysis program. *J. Appl. Cryst.* **42**, 339–341 (2009).

18. Sugimoto, K., Ohsumi, H., Aoyagi, S., Nishibori, E., Moriyoshi, C., Kuroiwa, Y., Sawa, H. & Takata, M. Extremely high resolution single crystal diffractometery for orbital resolution using high energy synchrotron radiation at SPring-8. *AIP Conf. Proc.* **1234**, 887–890 (2010).

19. CrysAlisPro, Agilent Technologies Ltd, Yarnton (2014).

20. Petříček, V., Dušek, M. & Palatinus, L. Discontinuous modulation functions and their application for analysis of modulated structures with the computing system JANA2006. *Z. Kristallogr. Cryst. Mater.* **229**, 345–352 (2014).

21. Buron-Le Cointe, M., Lemée-Cailleau, M. H., Cailleau, H., Ravy, S., Bérar, J. F., Rouziére, S., Elkaïm, E. & Collet, E. One-dimensional fluctuating nanodomains in the charge-transfer molecular system TTF-CA. *Phys. Rev. Lett.* **96**, 205503 (2006).

22. Kagoshima, S., Nagasawa, H. & Sambongi, T. *One-dimensional conductors*, pp. 48–105 (Springer-Verlag, 1988).

23. Momma, K. & Izumi, F. *VESTA3* for three-dimensional visualization of crystal, volumetric and morphology data. *J. Appl. Cryst*. **44**, 1272–1276 (2011).

24. Castagnetti, N., Masino, M., Rizzoli, C. & Girlando, A. Mixed stack charge transfer crystals: crossing the neutral-ionic borderline by chemical substitution. *Phys. Rev. Mater.* **2**, 024602 (2018).

25. Okamoto, H., Ishige, Y., Tanaka, S., Kishida, H., Iwai, S. & Tokura, Y. Photoinduced phase transition in tetrathiafulvalene-*p*-chloranil observed in femtosecond reflection spectroscopy. *Phys. Rev. B* **70**, 165202 (2004).

26. Rice, M. J. Organic linear conductors as systems for the study of electron-phonon interactions in the organic solid state. *J. Phys. Rev. Lett.* **37**, 36–39 (1976).

27. Girlando, A., Bozio, R., Pecile, C. & Torrance, J. B. Discovery of vibronic effects in the Raman spectra of mixed-stack charge-transfer crystals. *Phys. Rev. B* **26**, 2306–2309 (1982).

28. Painelli, A. & Girlando, A. Electron–molecular vibration (e–mv) coupling in charge-transfer compounds and its consequences on the optical spectra: A theoretical framework. *J. Chem. Phys.* **84**, 5655–5671 (1986).

29. Gaussian 16, Revision B.01, 2016. Gaussian, Inc., Wallingford CT, 2016. https://gaussian.com/.

30. Binning Jr. R. C. & Curtiss, L. A. Compact contracted basis sets for third-row atoms: Ga–Kr. *J. Comp. Chem.* **11**, 1206–1216 (1990).

31. Rassolov, V. A., Pople, J. A., Ratner, M. A. & Windus, T. L. 6-31G* basis set for atoms K through Zn. *J. Chem. Phys.* **109**, 1223–1229 (1998).

32. Rassolov, V. A., Ratner, M. A., Pople, J. A., Redfern, P. C. & Curtiss, L. A. 6-31G* basis set for third-row atoms. *J. Comp. Chem.* **22**, 976–984 (2001).

33. Mitani, T., Saito, G., Tokura, T. & Koda, T. Soliton formation at the neutral-to-ionic phase transition in the mixed-stack charge-transfer crystal tetrathiafulvalene-*p*-chloranil. *Phys. Rev. Lett.* **53**, 842–845 (1984).

34. Iwasa, Y., Koda, T., Tokura, Y., Kobayashi, A., Iwasawa, N. & Saito, G. Temperature-induced neutral-ionic transition in tetramethylbenzidine-tetracyanoquinodimethane (TMB-TCNQ). *Phys. Rev. B* **42**, 2374–2377 (1990).

35. Kagawa, F., Horiuchi, S., Tokunaga, M., Fujioka, J. & Tokura, Y. Ferroelectricity in a one-dimensional organic quantum magnet. *Nat. Phys.* **6**, 169–172 (2010).
